# How Attention Can Create Synaptic Tags for the Learning of Working Memories in Sequential Tasks

**DOI:** 10.1371/journal.pcbi.1004060

**Published:** 2015-03-05

**Authors:** Jaldert O. Rombouts, Sander M. Bohte, Pieter R. Roelfsema

**Affiliations:** 1 Department of Life Sciences, Centrum Wiskunde & Informatica, Amsterdam, The Netherlands; 2 Department of Vision & Cognition, Netherlands Institute for Neurosciences, an institute of the Royal Netherlands Academy of Arts and Sciences (KNAW), Amsterdam, The Netherlands; 3 Department of Integrative Neurophysiology, Centre for Neurogenomics and Cognitive Research, VU University, Amsterdam, The Netherlands; 4 Psychiatry Department, Academic Medical Center, Amsterdam, The Netherlands; École Normale Supérieure, College de France, CNRS, FRANCE

## Abstract

Intelligence is our ability to learn appropriate responses to new stimuli and situations. Neurons in association cortex are thought to be essential for this ability. During learning these neurons become tuned to relevant features and start to represent them with persistent activity during memory delays. This learning process is not well understood. Here we develop a biologically plausible learning scheme that explains how trial-and-error learning induces neuronal selectivity and working memory representations for task-relevant information. We propose that the response selection stage sends attentional feedback signals to earlier processing levels, forming synaptic tags at those connections responsible for the stimulus-response mapping. Globally released neuromodulators then interact with tagged synapses to determine their plasticity. The resulting learning rule endows neural networks with the capacity to create new working memory representations of task relevant information as persistent activity. It is remarkably generic: it explains how association neurons learn to store task-relevant information for linear as well as non-linear stimulus-response mappings, how they become tuned to category boundaries or analog variables, depending on the task demands, and how they learn to integrate probabilistic evidence for perceptual decisions.

## Author Summary

Working memory is a cornerstone of intelligence. Most, if not all, tasks that one can imagine require some form of working memory. The optimal solution of a working memory task depends on information that was presented in the past, for example choosing the right direction at an intersection based on a road-sign some hundreds of meters before. Interestingly, animals like monkeys readily learn difficult working memory tasks, just by receiving rewards such as fruit juice when they perform the desired behavior. Neurons in association areas in the brain play an important role in this process; these areas integrate perceptual and memory information to support decision-making. Some of these association neurons become tuned to relevant features and memorize the information that is required later as a persistent elevation of their activity. It is, however, not well understood how these neurons acquire their task-relevant tuning. Here we formulate a simple biologically plausible learning mechanism that can explain how a network of neurons can learn a wide variety of working memory tasks by trial-and-error learning. We also show that the solutions learned by the model are comparable to those found in animals when they are trained on similar tasks.

## Introduction

Animals like monkeys can be trained to perform complex cognitive tasks, simply by giving rewards at the right times. They can learn to map sensory stimuli onto responses, to store task-relevant information and to integrate and combine unreliable sensory evidence. Training induces new stimulus and memory representations in ‘multiple-demand’ regions of the cortex [1]. For example, if monkeys are trained to memorize the location of a visual stimulus, neurons in lateral intra-parietal cortex (LIP) represent this location as a persistent increase of their firing rate [2,3]. However, if the animals learn a visual categorization task, persistent activity of LIP cells becomes tuned to the boundary between categories [4] whereas the neurons integrate probabilistic evidence if the task is sensory decision making [5]. Similar effects of training on persistent activity have been observed in the somatosensory system. If monkeys are trained to compare frequencies of successive vibrotactile stimuli, working memory representations of analog variables are formed in somatosensory, prefrontal and motor cortex [6].

Which learning mechanism induces appropriate working memories in these tasks? We here outline AuGMEnT (Attention-Gated MEmory Tagging), a new reinforcement learning [7] scheme that explains the formation of working memories during trial-and-error learning and that is inspired by the role of attention and neuromodulatory systems in the gating of neuronal plasticity. AuGMEnT addresses two well-known problems in learning theory: temporal and structural credit-assignment [7,8]. The temporal credit-assignment problem arises if an agent has to learn actions that are only rewarded after a sequence of intervening actions, so that it is difficult to assign credit to the appropriate ones. AuGMEnT solves this problem like previous temporal-difference reinforcement learning (RL) theories [7]. It learns action-values (known as *Q*-values [7]), i.e. the amount of reward that is predicted for a particular action when executed in a particular state of the world. If the outcome deviates from the reward-prediction, a neuromodulatory signal that codes the global reward-prediction error (RPE) gates synaptic plasticity in order to change the *Q*-value, in accordance with experimental findings [9–12]. The key new property of AuGMEnT is that it can also learn tasks that require working memory, thus going beyond standard RL models [7,13].

AuGMEnT also solves the structural credit-assignment problem of networks with multiple layers. Which synapses should change to improve performance? AuGMEnT solves this problem with an ‘attentional’ feedback mechanism. The output layer has feedback connections to units at earlier levels that provide feedback to those units that were responsible for the action that was selected [14]. We propose that this feedback signal tags [15] relevant synapses and that the persistence of tags (known as eligibility traces [7,16]) permits learning if time passes between the action and the RPE [see 17]. We will here demonstrate the neuroscientific plausibility of AuGMEnT. A preliminary and more technical version of these results has been presented at a conference [18].

## Model

### Model architecture

**Fig 1:**
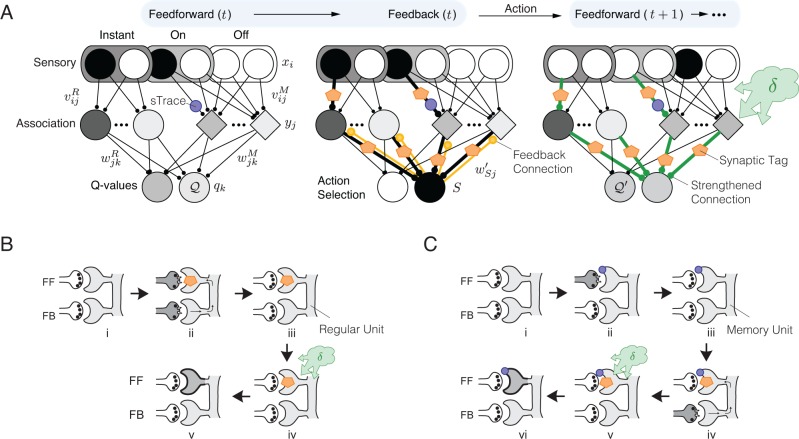
Model Architecture. ***A***, The model consists of a sensory input layer with units that code the input (instantaneous units) and transient units that only respond when a stimulus appears (on-units) or if it disappears (off-units). The association layer contains regular units (circles) with activities that depend on instantaneous input units, and integrating memory units (diamonds) that receive input from transient sensory units. The connections from the input layer to the memory cells maintain a synaptic trace (sTrace; blue circle) if the synapse was active. Units in the third layer code the value of actions (Q-values). After computing feed-forward activations, a Winner-Take-All competition determines the winning action (see middle panel). Action selection causes a feedback signal to earlier levels (through feedback connections $w_{\mathrm{Sj}}^{\prime }$, see middle panel) that lays down synaptic tags (orange pentagons) at synapses that are responsible for the selected action. If the predicted *Q*-value of the next action *S′* (*Q_S′_*) plus the obtained reward *r*(*t*) is higher than *Q_S_*, a globally released neuromodulator *δ* (see eq. (17)) interacts with the tagged synapses to increase the strength of tagged synapses (green connections). If the predicted value is lower than expected, the strength of tagged synapses is decreased. ***B***, Schematic illustration of the tagging process for regular units. FF is a feed-forward connection and FB is a feedback connection. The combination of feed-forward and feedback activation gives rise to a synaptic tag in step ii. Tags interact with the globally released neuromodulator *δ* to change the synaptic strength (step iv,v). ***C***, Tagging process for memory units. Any presynaptic feed-forward activation gives rise to a synaptic trace (step ii; sTrace—purple circle). A feedback signal from the *Q*-value unit selected for action creates synaptic tags on synapses that carry a synaptic trace (step iv). The neuromodulator can interact with the tags to modify synaptic strength (v,vi).

We used AuGMEnT to train networks composed of three layers of units connected by two layers of modifiable synapses (Fig. 1). Time was modeled in discrete steps.

### Input layer

At the start of every time step, feedforward connections propagate information from the sensory layer to the association layer through modifiable connections *v_ij_*. The sensory layer represents stimuli with instantaneous and transient units (Fig. 1). Instantaneous units represent the current sensory stimulus *x*(*t*) and are active as long as the stimulus is present. Transient units represent changes in the stimulus and behave like ‘on (+)’ and ‘off (-)’ cells in sensory cortices [19]. They encode positive and negative changes in sensory inputs w.r.t. the previous time-step *t* - 1:

$$x^{+}(t)={\left[x(t)-x(t-1)\right]}_{+}\;,$$ (1)

$$x^{-}(t)={\left[x(t-1)-x(t)\right]}_{+}\;,$$ (2)

where [⋅]_+_ is a threshold operation that returns 0 for all negative values, but leaves positive values unchanged. Every input is therefore represented by three sensory units. We assume that all units have zero activity at the start of the trial (*t* = 0), and that *t* = 1 at the first time-step of the trial.

### Association layer

The second (hidden) layer of the network models the association cortex, and contains regular units (circles in Fig. 1) and memory units (diamonds). We use the term ‘regular unit’ to reflect the fact that these are regular sigmoidal units that do not exhibit persistent activity in the absence of input. Regular units *j* are fully connected to instantaneous units *i* in the sensory layer by connections $v_{ij}^{R}$ (the superscript *R* indexes synapses onto regular units, and $v_{0j}^{R}$ is a bias weight). Their activity $y_{j}^{R}(t)$ is determined by:

$$inp_{j}^{R}(t)=\sum_{i}v_{ij}^{R}x_{i}(t),$$ (3)

$$y_{j}^{R}(t)=\sigma (inp_{j}^{R}(t)),$$ (4)

here $inp_{j}^{R}(t)$ denotes the synaptic input and *σ* a sigmoidal activation function;

$$\sigma (inp_{j}^{R}(t))=1/(1\text{​}+\text{exp}(\theta -inp_{j}^{R}(t))),$$ (5)

although our results do not depend on this particular choice of *σ*. The derivative of $y_{j}^{R}(t)$ can be conveniently expressed as:

$${y^{\prime }}_{j}^{R}(t)=\sigma^{\prime }(inp_{j}^{R}(t))=\frac{\partial y_{j}^{R}(t)}{\partial inp_{j}^{R}(t)}=y_{j}^{R}(t)(1-y_{j}^{R}(t)).$$ (6)

Memory units *m* (diamonds in Fig. 1) are fully connected to the transient (+/-) units in the sensory layer by connections $v_{lm}^{M}$ (superscript *M* indexes synapses onto memory units) and they integrate their input over the duration of the trial:

$$inp_{m}^{M}(t)=inp_{m}^{M}(t-1)+\sum_{l}v_{\text{l}m}^{M}x_{l}^{\prime }(t)\;,$$ (7)

$$y_{m}^{M}(t)=\sigma (inp_{m}^{M}(t))\;,$$ (8)

where we use the shorthand $x_{l}^{\prime }$ that stands for both + and - cells, so $\sum_{l}v_{\text{l}m}^{M}x_{l}^{\prime }(t)$ should be read as $\sum_{l}v_{\text{l}m}^{M+}x_{l}^{+}(t)+\sum_{l}v_{\text{l}m}^{M-}x_{l}^{-}(t)$ The selective connectivity between the transient input units and memory cells is advantageous. We found that the learning scheme is less stable when memory units also receive input from the instantaneous input units because in that case even weak constant input becomes integrated across time as an activity ramp. We note, however, that there are other neuronal mechanisms which can prevent the integration of constant inputs. For example, the synapses between instantaneous input units and memory units could be rapidly adapting, so that the memory units only integrate variations in their input.

The simulated integration process causes persistent changes in the activity of memory units. It is easy to see that the activity of a memory unit equals the activity of a hypothetical regular unit that would receive input from all previous time-steps of the trial at the same time. To keep the model simple, we do not simulate the mechanisms responsible for persistent activity, which have been addressed in previous work [20–22]. Although the perfect integration assumed in Eqn. (7) does not exist in reality, we suggest that it is an acceptable approximation for trials with a relatively short duration as in the tasks that will be described below. Indeed, there are reports of single neuron integrators in entorhinal cortex with stable firing rates that persist for ten minutes or more [23], which is orders of magnitude longer than the trials modeled here. In neurophysiological studies in behaving animals, the neurons that behave like regular and memory units in e.g. LIP [2,3] and frontal cortex [24] would be classified as visual cells and memory cells, respectively.

### Q-value layer

The third layer receives input from the association layer through plastic connections *w*_jk_ (Fig. 1). Its task is to compute action-values (i.e. *Q*-values [7]) for every possible action. Specifically, a *Q*-value unit aims to represent the (discounted) expected reward for the remainder of a trial if the network selects an action *a* in the current state *s* [7]:

$$Q^{\pi }(s,a)=E_{\pi }\left\{R_{t}|s_{t}=s,a_{t}=a\right\},\;\text{with}\;R_{t}=\sum_{p=0}^{\infty }\gamma^{p}r_{t+p+1}\;,$$ (9)

where the $E_{\pi }\left\{\cdot \right\}$ term is the expected discounted future reward *R_t_* given *a* and *s*, under action-selection policy *π* and γ∈[0,1] determines the discounting of future rewards *r*. It is informative to explicitly write out the above expectation to see that *Q*-values are recursively defined as:

$$Q^{\pi }(s,a)=\sum_{s^{\prime }\in S}P_{sa}^{s\prime }\left[R_{sa}^{s\prime }+\gamma \sum_{a^{\prime }\in A}\pi (a^{\prime }|s^{\prime })Q^{\pi }(s^{\prime },a^{\prime })\right],$$ (10)

where $P_{sa}^{s\prime }$ is a transition matrix, containing the probabilities that executing action *a* in state *s* will move the agent to state *s*′, $R_{sa}^{s\prime }$ is the expected reward for this transition, and *S* and *A* are the sets of states and actions, respectively. Note that the action selection policy *π* is assumed to be stochastic in general. By executing the policy *π*, an agent samples trajectories according to the probability distributions *π*, $P_{sa}^{s\prime }$ and $R_{sa}^{s\prime }$ where every observed transition can be used to update the original prediction *Q*(*s_t_*, *a_t_*). Importantly, temporal difference learning schemes such as AuGMEnT are *model-free*, which means that they do not need explicit access to these probability distributions while improving their *Q*-values.

*Q*-value units *k* are fully connected to the association layer by connections $w_{jk}^{R}$ (from regular units, with $w_{0k}^{R}$ as bias weight) and $w_{mk}^{M}$ (from memory units). The action value *q_k_*(*t*) is estimated as:

$$q_{k}(t)=\sum_{m}w_{mk}^{M}y_{m}^{M}(t)+\sum_{j}w_{jk}^{R}y_{j}^{R}(t)\;,$$ (11)

where *q_k_*(*t*) aims to represent the value of action *k* at time step *t*, i.e. if *a_t_* = *k*. In AuGMEnT, the state *s* in Eq. (9) is represented by the vector of activations in the association layer. Association layer units must therefore learn to represent and memorize information about the environment to compute the value of all possible actions *a*. They transform a so-called partially observable Markov decision process (POMDP) where the optimal decision depends on information presented in the past into a simpler Markov decision process (MDP) by storing relevant information as persistent activity, making it available for the next decision.

### Action selection

The action-selection policy *π* is implemented by a stochastic winner-takes-all (WTA) competition biased by the *Q*-values. The network usually chooses the action *a* with the highest value, but occasionally explores other actions to improve its value estimates. We used a Max-Boltzmann controller [25] to implement the action selection policy *π*. It selects the greedy action (highest *q_k_*(*t*), ties are broken randomly) with probability 1 - *ε*, and a random action *k* sampled from the Boltzmann distribution *P_B_* with small probability *ε*:

$$P_{B}(k)=\frac{\text{exp}(q_{k})}{\sum_{k^{\prime }}\text{exp}(q_{k^{\prime }})}\;.$$ (12)

This controller ensures that the model explores all actions, but usually selects the one with the highest expected value. We assume that the controller is implemented downstream, e.g. in the motor cortex or basal ganglia, but do not simulate the details of action selection, which have been addressed previously [26–30]. After selecting an action *a*, the activity in the third layer becomes *z_k_* = *δ_ka_*, where *δ_ka_* is the Kronecker delta function (1 if *k* = *a* and 0 otherwise). In other words, the selected action is the only one active after the selection process, and it then provides an “attentional” feedback signal to the association cortex (orange feedback connections in Fig. 1A).

### Learning

Learning in the network is controlled by two factors that gate plasticity: a global neuromodulatory signal (described below) and the attentional feedback signal. Once an action is selected, the unit that codes the winning action *a* feeds back to earlier processing levels to create synaptic tags [31,32], also known as eligibility traces [7,16] on the responsible synapses (orange pentagons in Fig. 1). Tagging of connections from the association layer to the motor layer follows a form of Hebbian plasticity: the tag strength depends on presynaptic activity (*y_j_*) and postsynaptic activity *after* action selection (*z_k_*) and tags thus only form at synapses *w_ja_* onto the winning (i.e. selected) motor unit *a*:

$$\begin{array}{l} \Delta Tag_{jk}=-\alpha Tag_{jk}+y_{j}z_{k}\;,\text{which is equivalent to:}\\ \Delta Tag_{ja}=-\alpha Tag_{ja}+y_{j}\;,\text{for the winning action}\;a,\;\text{because}\;z_{a}=1\;\text{and}\\ \Delta Tag_{jk}=-\alpha Tag_{jk}\;,\text{for}\;k\neq a,\;\text{because}\;z_{k\neq a}=0, \end{array}$$ (13)

where *α* controls the decay of tags. Here, Δ denotes the change in one time-step, i.e *Tag*(*t*+1) = *Tag*(*t*)+Δ*Tag*(*t*).

The formation of tags on the feedback connections $w_{aj}^{\prime }$ follows the same rule so that the strength of feedforward and feedback connections becomes similar during learning, in accordance with neurophysiological findings [33]. Thus, the association units that provided strong input to the winning action *a* also receive strongest feedback (Fig. 1, middle panel): they will be held responsible for the outcome of *a*. Importantly, the attentional feedback signal also guides the formation of tags on connections *v_ij_* so that synapses from the input layer onto responsible association units *j* (strong $w_{aj}^{\prime }$) are most strongly tagged (Fig. 1B).

For regular units we propose:

$$\Delta Tag_{ij}\;=-\alpha Tag_{ij}+x_{i}\sigma^{\prime }(inp_{j})w_{aj}^{\prime }\;,$$ (14)

where *σ*′ is the derivative of the association unit’s activation function *σ* (Eq. (5)), which determines the influence that a change in the input *inp_j_* has on the activity of unit *j*. The idea has been illustrated in Fig. 1B. Feedback from the winning action (lower synapse in Fig. 1B) enables the formation of tags on the feedforward connections onto the regular unit. These tags can interact with globally released neuromodulators that inform all synapses about the RPE (green cloud ‘*δ*’ in Fig. 1). Note that feedback connections only influence the plasticity of representations in the association layer but do not influence activity in the present version of the model. We will come back to this point in the discussion.

In addition to synaptic tags, AuGMEnT uses synaptic traces (sTrace, blue circle in Fig. 1A,C) for the learning of new working memories. These traces are located on the synapses from the sensory units onto memory cells. Any pre-synaptic activity in these synapses leaves a trace that persists for the duration of a trial. If one of the selected actions provides a feedback signal (panel iv in Fig. 1C) to the post-synaptic memory unit, the trace gives rise to a tag making the synapse plastic as it can now interact with globally released neuromodulators:

$$\Delta sTrace_{ij}=x_{i}\;,$$ (15)

$$\Delta Tag_{ij}\;=-\alpha Tag_{ij}+sTrace_{ij}\sigma^{\prime }(inp_{j})w_{aj}^{\prime }$$ (16)

We assume that the time scale of trace updates is fast compared to the tag updates, so that tags are updated with the latest traces. The traces persist for the duration of the trial, but all tags decay exponentially (0<*α*<1).

After executing an action, the network may receive a reward *r*(*t*). Moreover, an action *a* at time step (*t*-1) may have caused a change in the sensory stimulus. For example, in most studies of monkey vision, a visual stimulus appears if the animal directs gaze to a fixation point. In the model, the new stimulus causes feedforward processing on the next time step *t*, which results in another set of *Q*-values. To evaluate whether *a* was better or worse than expected, the model compares the predicted outcome *Q_a_*(*t*-1), which has to be temporarily stored in the system, to the sum of the reward *r*(*t*) and the discounted action-value *Q_a′_*(*t*) of unit *a′* that wins the subsequent stochastic WTA-competition. This temporal difference learning rule is known as SARSA [7,34]:

$$\delta (t)\;=r(t)+\gamma q_{a^{\prime }}(t)-q_{a}(t-1)\;.$$ (17)

The RPE *δ*(*t*) is positive if the outcome of *a* is better than expected and negative if it is worse. Neurons representing action values have been found in the frontal cortex, basal ganglia and midbrain [12,35,36] and some orbitofrontal neurons specifically code the chosen value, *q_a_* [37]. Moreover, dopamine neurons in the ventral tegmental area and substantia nigra represent *δ* [9,10,38]. In the model, the release of neuromodulators makes *δ* available throughout the brain (green cloud in Fig. 1).

Plasticity of all synapses depends on the product of *δ* and tag strength:

$$\begin{array}{c} \Delta v_{ij}=\beta \delta (t)Tag_{ij}\;,\\ \Delta w_{jk}=\beta \delta (t)Tag_{jk}\;, \end{array}$$ (18)

where *β* is the learning rate, and where the latter equation also holds for the feedback weights $w_{kj}^{\prime }$. These equations capture the key idea of AuGMEnT: tagged synapses are held accountable for the RPE and change their strength accordingly. Note that AuGMEnT uses a four-factor learning rule for synapses *v_ij_*. The first two factors are the pre- and postsynaptic activity that determine the formation of tags (Eqns. (14)–(16)). The third factor is the “attentional” feedback from the motor selection stage, which ensures that tags are only formed in the circuit that is responsible for the selected action. The fourth factor is the RPE *δ*, which reflects whether the outcome of an action was better or worse than expected and determines if the tagged synapses increase or decrease in strength. The computation of the RPE demands the comparison of *Q*-values in different time-steps. The RPE at time *t* depends on the action that the network selected at *t*-1 (see Eqn. (17) and the next section), but the activity of the units that gave rise to this selection have typically changed at time *t*. The synaptic tags solve this problem because they labeled those synapses that were responsible for the selection of the previous action.

AuGMEnT is biologically plausible because the equations that govern the formation of synaptic tags (Eqns. (13), (14), (16)) and traces (Eq. (15)) and the equations that govern plasticity (Eq. (18)) rely only on information that is available locally, at the synapse. Furthermore, the hypothesis that a neuromodulatory signal, like dopamine, broadcasts the RPE to all synapses in the network is supported by neurobiological findings [9,10,38].

## Results

We will now present the main theoretical result, which is that the AuGMEnT learning rules minimize the temporal difference errors (Eqn. (17)) of the transitions that are experienced by the network by on-line gradient descent. Although AuGMEnT is not guaranteed to find optimal solutions (we cannot provide a proof of convergence), we found that it reliably learns difficult non-linear working memory problems, as will be illustrated below.

### AuGMEnT minimizes the reward-prediction error (RPE)

The aim of AuGMEnT is to reduce the RPE *δ*(*t*) because low RPEs for all network states imply reliable *Q*-values so that the network can choose the action that maximizes reward at every time-step. The RPE *δ*(*t*) implies a comparison between two quantities: the *predicted Q*-value before the transition, *q_a_*(*t*-1), and a *target Q*-value *r*(*t*)+*γq_a′_*(*t*), which consists of the actually observed reward and the next predicted *Q*-value [7]. If the two terms cancel, the prediction was correct. SARSA aims to minimize the prediction error by adjusting the network weights *w* to improve the prediction *q_a_*(*t*-1) to bring it closer to the observed value *r*(*t*)+*γq_a′_*(*t*). It is convenient to do this through on-line gradient descent on the squared prediction error $E(q_{a}(t-1))=\frac{1}{2}{\left(\left[r(t)+\gamma q_{a^{\prime }}(t)\right]-q_{a}(t-1)\right)}^{2}$ with respect to the parameters *w* [7,34]:

$$\Delta w\propto -\frac{\partial E(q_{a}(t-1))}{\partial w}=-\frac{\partial E(q_{a}(t-1))}{\partial q_{a}(t-1)}\frac{\partial q_{a}(t-1)}{\partial w}=\delta (t)\frac{\partial q_{a}(t-1)}{\partial w}\;,$$ (19)

where $\frac{\partial q_{a}(t-1)}{\partial w}$ is the gradient of the predicted *Q*-value *Q_a_*(*t*-1) with respect to parameters *w*. In Equation (19) we have used $\delta (t)=-\frac{\partial E(q_{a}(t-1))}{\partial q_{a}(t-1)}$, which follows from the definition of *E*(*q_a_*(*t*-1)). Note that *E* is defined with regard to the sampled transition only so that the definition typically differs between successive transitions experienced by the network. For notational convenience we will abbreviate *E*(*q_a_*(*t*-1)) to *E_q_a__* in the remainder of this paper.

We will refer to the negative of Equation (19) as “error gradient” in the remainder of this paper. The RPE is high if the sum of the reward *r*(*t*) and discounted *q_a′_*(*t*) deviates strongly from the prediction *q_a_*(*t*-1) on the previous time step. As in other SARSA methods, the updating of synaptic weights is only performed for the transitions that the network actually experiences. In other words, AuGMEnT is a so-called “on policy” learning method [7].

We will first establish the equivalence of on-line gradient descent defined in Equation (19) and the AuGMEnT learning rule for the synaptic weights $w_{jk}^{R}(t)$ from the regular units onto the *Q*-value units (Fig. 1). According to Equation (19), weights $w_{ja}^{R}$ for the chosen action *k* = *a* on time step *t*-1 should change as:

$$\Delta w_{ja}^{R}\;\propto \delta (t)\frac{\partial q_{a}(t-1)}{\partial w_{ja}^{R}(t-1)}\;,$$ (20)

leaving the other weights *k*≠*a* unchanged.

We will now show that AuGMEnT causes equivalent changes in synaptic strength. It follows from Eq. (11) that the influence of $w_{ja}^{R}$ on *q_a_*(*t*-1) (i.e. $\frac{\partial q_{a}(t-1)}{\partial w_{ja}^{R}(t-1)}$ in Eq. (20)) equals $y_{j}^{R}(t-1)$, the activity of association unit *j* on the previous time step. This result allows us to rewrite (20) as:

$$\Delta w_{ja}^{R}\propto -\frac{\partial E_{q_{a}}}{\partial w_{ja}^{R}(t-1)}\;=\delta (t)\;\frac{\partial q_{a}(t-1)}{\partial w_{ja}^{R}(t-1)}=\delta (t)y_{j}^{R}(t-1)\;.$$ (21)

Recall from Eq. (13) that the tags on synapses onto the winning output unit *a* are updated according to Δ*Tag_ja_* = - *αTag_ja_*+*y_j_* (orange pentagons in Fig. 1). In the special case *α* = 1, it follows that on time step *t*, $Tag_{ja}(t)=y_{j}^{R}(t-1)$ and that tags on synapses onto output units *k*≠*a* are 0. As a result,

$$\Delta w_{ja}^{R}\propto \delta (t)y_{j}^{R}(t-1)\;=\delta (t)Tag_{ja}(t)\;,$$ (22)

$$=\delta (t)Tag_{jk}(t)\;,$$ (23)

for the synapses onto the selected action *a*, and the second, generalized, equation follows from the fact that $\frac{\partial q_{k}(t-1)}{\partial w_{jk}^{R}(t-1)}=0$ for output units *k*≠*a* that were not selected and therefore do not contribute to the RPE. Inspection of Eqns. (18) and (23) reveals that AuGMEnT indeed takes a step of size *β* in the direction opposite to the error gradient of Equation (19) (provided *α* = 1; we discuss the case *α*≠1 below).

The updates for synapses between memory units *m* and *Q*-value units *k* are equivalent to those between regular units and the *Q*-value units. Thus,

$$\Delta w_{mk}^{M}\;\propto -\frac{\partial E_{q_{a}}}{\partial w_{mk}^{M}(t-1)}=\delta (t)\;\frac{\partial q_{k}(t-1)}{\partial w_{mk}^{M}(t-1)}=\delta (t)Tag_{mk}(t).$$ (24)

The plasticity of the feedback connections ${w^{\prime }}_{kj}^{R}$ and ${w^{\prime }}_{km}^{M}$ from the *Q*-value layer to the association layer follows the same rule as the updates of connections $w_{jk}^{R}$ and $w_{mk}^{M}$ and the feedforward and feedback connections between two units therefore become proportional during learning [14].

We will now show that synapses $v_{ij}^{R}$ between the input layer and the regular association units (Fig. 1) also change according to the negative gradient of the error function defined above. Applying the chain rule to compute the influence of $v_{ij}^{R}$ on *q_a_*(*t*-1) results in the following equation:

$$\begin{array}{c} \Delta v_{ij}^{R}\propto \delta (t)\frac{\partial q_{a}(t-1)}{\partial y_{j}^{R}(t-1)}\frac{\partial y_{j}^{R}(t-1)}{\partial inp_{j}^{R}(t-1)}\frac{\partial inp_{j}^{R}(t-1)}{\partial v_{ij}^{R}(t-1)}\;,\\ =\delta (t)w_{ja}^{R}\sigma^{\prime }(inp_{j}^{R}(t-1))x_{i}(t-1)\;. \end{array}$$ (25)

The amount of attentional feedback that was received by unit *j* from the selected *Q*-value unit *a* at time *t*-1 is equal to ${w^{\prime }}_{aj}^{R}$ because the activity of unit *a* equals 1 once it has been selected. As indicated above, learning makes the strength of feedforward and feedback connections similar so that $w_{ja}^{R}$ can be estimated as the amount of feedback ${w^{\prime }}_{aj}^{R}$ that unit *j* receives from the selected action *a*,

$$\Delta v_{ij}^{R}\propto -\frac{\partial E_{q_{a}}}{\partial v_{ij}^{R}(t-1)}\;=\delta (t){w^{\prime }}_{aj}^{R}\sigma^{\prime }(inp_{j}^{R}(t-1))x_{i}(t-1)\;.$$ (26)

Recall from Eq. (14) that the tags on synapses $v_{ij}^{R}$ are updated according to $\Delta Tag_{ij}=-\alpha Tag_{ij}+x_{i}\sigma^{\prime }(inp_{j}){w^{\prime }}_{aj}^{R}$. Fig. 1B illustrates how feedback from action *a* controls the tag formation process. If *α* = 1, then on time step *t*, $Tag_{ij}(t)=x_{i}(t-1)\sigma^{\prime }(inp_{j}^{R}(t-1)){w^{\prime }}_{aj}^{R}$ so that Eq. (26) can be written as:

$$\Delta v_{ij}^{R}\propto -\frac{\partial E_{q_{a}}}{\partial v_{ij}^{R}(t-1)}=\delta (t)Tag_{ij}(t)\;.$$ (27)

A comparison to Eq. (18) demonstrates that AuGMEnT also takes a step of size *β* in the direction opposite to the error gradient for these synapses.

The final set of synapses that needs to be considered are between the transient sensory units and the memory units. We approximate the total input $inp_{m}^{M}(t)$ of memory unit *m* as (see Eq. (7)):

$$\begin{array}{c} inp_{m}^{M}(t)=\sum_{l}v_{lm}^{M}(t)x_{l}^{\prime }(t)+\sum_{l,t^{\prime }=0}^{t-1}v_{lm}^{M}(t^{\prime })x_{l}^{\prime }(t^{\prime })\;,\\ \approx \sum_{l}v_{lm}^{M}(t)\sum_{t^{\prime }=0}^{t}x_{l}^{\prime }(t^{\prime })\;, \end{array}$$ (28)

The approximation is good if synapses $v_{lm}^{M}$ change slowly during a trial. According to Equation (19), the update for these synapses is:

$$\begin{array}{c} \Delta v_{lm}^{M}\propto -\frac{\partial E_{q_{a}}}{\partial v_{lm}^{M}(t-1)}=\delta (t)\frac{\partial q_{a}(t-1)}{\partial y_{m}^{M}(t-1)}\frac{\partial y_{m}^{M}(t-1)}{\partial inp_{m}^{M}(t-1)}\frac{\partial inp_{m}^{M}(t-1)}{\partial v_{lm}^{M}(t-1)}\;,\\ =\delta (t){w^{\prime }}_{am}^{M}\sigma^{\prime }(inp_{m}^{M}(t-1))[\sum_{t^{\prime }=0}^{t-1}x_{l}^{\prime }(t^{\prime })]\;. \end{array}$$ (29)

Eq. (15) specifies that Δ*sTrace_lm_* = *x_l_* so that $sTrace_{lm}(t-1)=\sum_{t^{\prime }=0}^{t-1}x_{l}^{\prime }(t^{\prime })$, the total presynaptic activity of the input unit up to time *t*-1 (blue circle in Fig. 1C). Thus, Eq. (29) can also be written as:

$$\Delta v_{lm}^{M}\propto \delta (t){w^{\prime }}_{am}^{M}\sigma^{\prime }(inp_{m}^{M}(t-1))sTrace_{lm}(t-1)\;.$$ (30)

Eq. (16) states that $\Delta Tag_{lm}=-\alpha Tag_{lm}+sTrace_{lm}\sigma^{\prime }(inp_{m}^{M}){w^{\prime }}_{am}^{M}$, because the feedback from the winning action *a* converts the trace into a tag (panel iv in Fig. 1C). Thus, if *α* = 1 then $Tag_{lm}^{M}(t)={w^{\prime }}_{am}^{M}\sigma^{\prime }(inp_{m}^{M}(t-1))sTrace_{lm}(t-1)$ so that:

$$\Delta v_{lm}^{M}\propto \delta (t)Tag_{lm}^{M}(t).$$ (31)

Again, a comparison of Eqns. (31) and (18) shows that AuGMEnT takes a step of size *β* in the direction opposite to the error gradient, just as is the case for all other categories of synapses. We conclude that AuGMEnT causes an on-line gradient descent on all synaptic weights to minimize the temporal difference error if *α* = 1.

AuGMEnT provides a biological implementation of the well known RL method called SARSA, although it also goes beyond traditional SARSA [7] by (i) including memory units (ii) representing the current state of the external world as a vector of activity at the input layer (iii) providing an association layer that aids in computing *Q*-values that depend non-linearly on the input, thus providing a biologically plausible equivalent of the error-backpropagation learning rule [8], and (iv) using synaptic tags and traces (Fig. 1B,C) so that all the information necessary for plasticity is available locally at every synapse.

The tags and traces determine the plasticity of memory units and aid in decreasing the RPE by improving the *Q*-value estimates. If a memory unit *j* receives input from input unit *i* then a trace of this input is maintained at synapse *v_ij_* for the remainder of the trial (blue circle in Fig. 1C). Suppose that *j*, in turn, is connected to action *a* which is selected at a later time point. Now unit *j* receives feedback from *a* so that the trace on synapse *v_ij_* becomes a tag making it sensitive to the globally released neuromodulator that codes the RPE *δ* (panel iv in Fig. 1C). If the outcome of *a* was better than expected (*δ*>0) (green cloud in panel v), *v_ij_* strengthens (thicker synapse in panel vi). When the stimulus that activated unit *i* reappears on a later trial, the larger *v_ij_* increases unit *j* ’s persistent activity which, in turn, enhances the activity of the *Q*-value unit representing *a*, thereby decreasing the RPE.

The synaptic tags of AuGMEnT correspond to the eligibility traces used in RL schemes. In SARSA learning speeds up if the eligibility traces do not fully decay on every time step, but exponentially with parameter λ∈[0,1] [7]; the resulting rule is called SARSA(λ). In AuGMEnT, the parameter *α* plays an equivalent role and precise equivalence can be obtained by setting *α* = 1-λ*γ* as can be verified by making this substitution in Eqn. (13) (14) and (16) (noting that *Tag*(*t*+1) = *Tag*(*t*)+Δ*Tag*(*t*)). It follows that tags decay exponentially as *Tag*(*t*+1) = λ*γTag*(t), equivalent to the decay of eligibility traces in SARSA(λ). These results establish the correspondence between the biologically inspired AuGMEnT learning scheme and the RL method SARSA(λ). A special condition occurs at the end of a trial. The activity of memory units, traces, tags, and *Q*-values are set to zero (see [7]), *after* updating of the weights with a *δ* that reflects the transition to the terminal state.

In the remainder of the results section we will illustrate how AuGMEnT can train multi-layered networks with the form of Fig. 1 to perform a large variety of tasks that have been used to study neuronal representations in the association cortex of monkeys.

### Using AuGMEnT to simulate animal learning experiments

We tested AuGMEnT on four different tasks that have been used to investigate the learning of working memory representations in monkeys. The first three tasks have been used to study the influence of learning on neuronal activity in area LIP and the fourth task to study vibrotactile working memory in multiple cortical regions. All tasks have a similar overall structure: the monkey starts a trial by directing gaze to a fixation point or by touching a response key. Then stimuli are presented to the monkey and it has to respond with the correct action after a memory delay. At the end of a trial, the model could choose between two possible actions. The full task reward (*r_f_*, 1.5 units) was given if this choice was correct, while we aborted trials and gave no reward if the model made the wrong choice or broke fixation (released the key) before a go signal.

Researchers usually train monkeys on these tasks with a shaping strategy. The monkey starts with simple tasks and then the complexity is gradually increased. It is also common to give small rewards for reaching intermediate goals in the task, such as attaining fixation. We encouraged fixation (or touching the key in the vibrotactile task below) by giving a small shaping reward (*r_i_*, 0.2 units) if the model directed gaze to the fixation point (touched the key). In the next section we will demonstrate that the training of networks with AuGMEnT is facilitated by shaping. Shaping was not necessary for learning in any of the tasks, however, but it enhanced learning speed and increased the proportion of networks that learned the task within the alloted number of training trials.

Across all the simulations, we used a single, fixed configuration of the association layer (three regular units, four memory units) and *Q*-layer (three units) and a single set of learning parameters (Tables 1,2). The number of input units varied across tasks as the complexity of the sensory stimuli differed. We note, however, that the results described below would have been identical had we simulated a fixed, large input layer with silent input units in some of the tasks, because silent input units have no influence on activity in the rest of the network.

**Table 1:** Model parameters.

Parameter	Description	Value
*β*	Learning rate	0.15
λ	Tag/Trace decay rate	0.20
*γ*	Discount factor	0.90
*α*	Tag persistence	1-λ*γ*
*ε*	Exploration rate	0.025

**Table 2:** Network architecture parameters.

Architecture	Value
**Input units**	Task dependent
**Memory units**	N = 4
**Regular units**	N = 3
**Q-value units**	N = 3
**Initial weights**	Uniform over [-0.25,0.25]

### Saccade/antisaccade task

**Fig 2:**
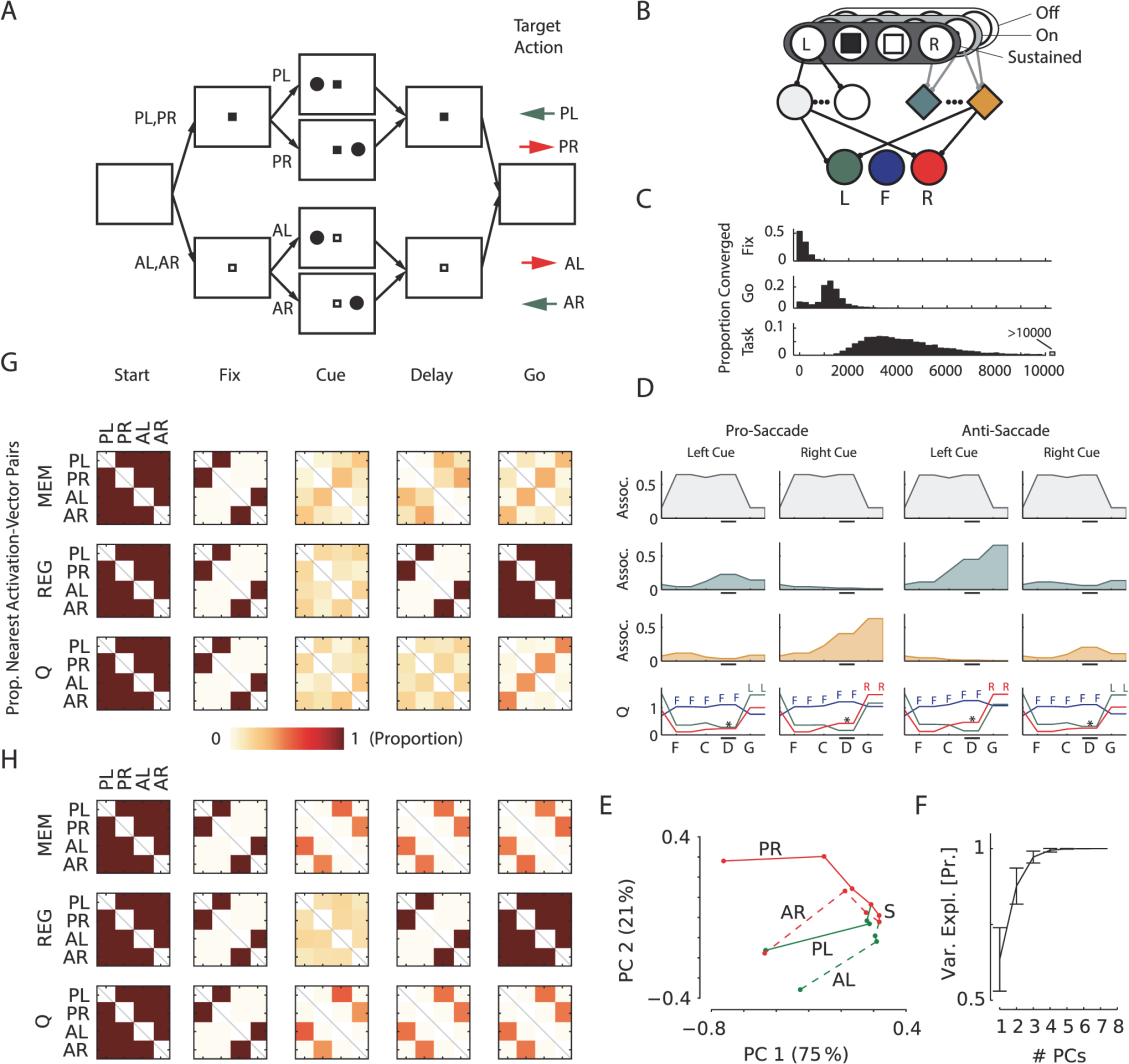
Saccade/antisaccade task. ***A***, Structure of the task, all possible trials have been illustrated. Fixation mark color indicates whether a saccade (P) or anti-saccade (A) is required after a memory delay. Colored arrows show the required action for the indicated trial types. L: cue left; R: cue right. ***B***, The sensory layer represents the visual information (fixation point, cue left/right) with sustained and transient (on/off) units. Units in the *Q*-value layer code three possible eye positions: left (green), center (blue) and right (red). ***C***, Time course of learning: 10,000 networks were trained, of which 9,945 learned the task within 25,000 trials. Histograms show the distribution of trials when the model learned to fixate (‘fix’), maintain fixation until the ‘go’-signal (‘go’) and learned the complete task (‘task’). ***D***, Activity of example units in the association and Q-layer. The grey trace illustrates a regular unit and the green and orange traces memory units. The bottom graphs show activity of the Q-value layer cells. Colored letters denote the action with highest *Q*-value. Like the memory cells, *Q*-value units also have delay activity that is sensitive to cue location (* in the lower panel) and their activity increases after the go-signal. ***E***, 2D-PCA projection of sequence of association layer activations for the four different trial types for an example network. S marks the start of the trials (empty screen). Pro saccade trials are shown with solid lines and anti-saccade trials with dashed lines. Color indicates cue location (green – left; red – right) and labels indicate trial type (P/A = type pro/anti; L/R = cue left/right). Percentages on the axes show variance explained by the PCs. ***F***, Mean variance explained as a function of the number of PCs over all 100 trained networks, error bars s.d. ***G***, Pairwise analysis of activation vectors of different unit types in the network (see main text for explanation). MEM: memory; REG: regular. This panel is aligned with the events in panel (A). Each square within a matrix indicates the proportion of networks where the activity vectors of different trial types were most similar. Color scale is shown below. For example, the right top square for the memory unit matrix in the ‘go’ phase of the task indicates that around 25% of the networks had memory activation vectors that were most similar for Pro-Left and Anti-Right trials. ***H***, Pairwise analysis of activation-vectors for networks trained on a version of the task where only pro-saccades were required. Conventions as in (G).

The first task (Fig. 2A) is a memory saccade/anti-saccade task modeled after Gottlieb and Goldberg [3]. Every trial started with an empty screen, shown for one time step. Then a fixation mark was shown that was either black or white, indicating that a pro- or anti-saccade would be required. The model had to fixate within 10 time-steps, otherwise the trial was terminated without reward. If the model fixated for two time-steps, we presented a cue on the left or the right side of the screen for one time-step and gave the fixation reward *r*_i_. This was followed by a memory delay of two time steps during which only the fixation point was visible. At the end of the memory delay the fixation mark turned off. To collect the final reward *r*_f_ in the pro-saccade condition, the model had to make an eye-movement to the remembered location of the cue and to the opposite location on anti-saccade trials. The trial was aborted if the model failed to respond within eight time steps.

The input units of the model (Fig. 2B) represented the color of the fixation point and the presence of the peripheral cues. The three *Q*-value units had to represent the value of directing gaze to the centre, left and right side of the screen. This task can only be solved by storing cue location in working memory and, in addition, requires a non-linear transformation and can therefore not be solved by a linear mapping from the sensory units to the *Q*-value units. We trained the models for maximally 25,000 trials, or until they learned the task. We kept track of accuracy for all four trial types as the proportion correct responses in the last 50 trials. When all accuracies reached 0.9 or higher, learning and exploration were disabled (i.e. *β* and *ε* were set to zero) and we considered learning successful if the model performed all trial-types accurately.

We found that learning of this task with AuGMEnT was efficient. We distinguished three points along the task learning trajectory: learning to obtain the fixation reward (‘Fix’), learning to fixate until fixation-mark offset (‘Go’) and finally to correctly solve the task (‘Task’). To determine the ‘Fix’-learn trial, we determined the time point when the model attained fixation in 90 out of 100 consecutive trials. The model learned to fixate after 224 trials (median) (Fig. 2C). The model learned to maintain gaze until the go signal after ∼1,300 trials and it successfully learned the complete task after ∼4,100 trials. Thus, the learning process was at least an order of magnitude faster than in monkeys that typically learn such a task after months of training with more than 1,000 trials per day.

To investigate the effect of the shaping strategy, we also trained 10,000 networks without the extra fixation reward (*r_i_* was zero). Networks that received fixation rewards were more likely to learn than networks that did not (99.45% versus 76.41%; *χ* ^2^ = 2,498, *p*<10^-6^). Thus, shaping strategies facilitate training with AuGMEnT, similar to their beneficial effect in animal learning [39].

The activity of a fully trained network is illustrated in Fig. 2D. One of the association units (grey in Fig. 2D) and the *Q*-unit for fixating at the centre of the display (blue in Fig. 2B,D) had strongest activity at fixation onset and throughout the fixation and memory delays. If recorded in a macaque monkey, these neurons would be classified as fixation cells. After the go-signal the *Q*-unit for the appropriate eye movement became more active. The activity of the *Q*-units also depended on cue-location during the memory delay as is observed, for example, in the frontal eye fields (* in Fig. 2D) [40]. This activity is caused by the input from memory units in the association layer that memorized cue location as a persistent increase in their activity (green and orange in Fig. 2D). Memory units were also tuned to the color of the fixation mark which differentiated pro-saccade trials from anti-saccade trials, a conjoined selectivity necessary to solve this non-linear task [41]. There was an interesting division of labor between regular and memory units in the association layer. Memory units learned to remember the cue location. In contrast, regular units learned to encode the presence of task-relevant sensory information on the screen. Specifically, the fixation unit in Fig. 2D (upper row) was active as long as the fixation point was present and switched off when it disappeared, thus cueing the model to make an eye movement. Interestingly, these two classes of memory neurons and regular (“light sensitive”) neurons are also found in areas of the parietal and frontal cortex of monkeys [2,40] where they appear to have equivalent roles.

Fig. 2D provides a first, casual impression of the representations that the network learns. To gain a deeper understanding of the representation in the association layer that supports the non-linear mapping from the sensory units to the *Q*-value units, we performed a principal component analysis (PCA) on the activations of the association units. We constructed a single (32x7) observation matrix from the association layer activations for each time-step (there were seven association units and eight time-points in each of the four trial-types), with the learning rate *β* and exploration rate *ε* of the network set to zero. Fig. 2E shows the projection of the activation vectors onto the first two principal components for an example network. It can be seen activity in the association layer reflects the important events in the task. The color of the fixation point and the cue location provide information about the correct action and lead to a ‘split’ in the 2D principal component (PC) space. In the ‘Go’ phase, there are only two possible correct actions: ‘left’ for the Pro-Left and Anti-Right trials and ‘right’ otherwise. The 2D PC plot shows that the network splits the space into three parts based on the optimal action: here the ‘left’ action is clustered in the middle, and the two trial types with target action ‘right’ are adjacent to this cluster. This pattern (or its inversion with the ‘right’ action in the middle) was typical for the trained networks. Fig. 2F shows how the explained variance in the activity of association units increases with the number of PCs, averaged over 100 simulated networks; most variance was captured by the first two PCs.

To investigate the representation that formed during learning across all simulated networks, we next evaluated the similarity of activation patterns (Euclidean distance) across the four trial types for the regular and memory association units and also for the units in the *Q*-value layer (Fig. 2G). For every network we entered a ‘1’ in the matrix for trial types with the smallest distance and a ‘0’ for all other pairs of trials and then aggregated results over all networks by averaging the resulting matrices. Initially the patterns of activity in the association layer are similar for all trial types, but they diverge after the presentation of the fixation point and the cue. The regular units convey a strong representation of the color of the fixation point (e.g. activity in pro-saccade trials with a left cue is similar to activity in pro-saccade trials with a right cue; PL and PR in Fig. 2G), which is visible at all times. Memory units have a clear representation of the previous cue location during the delay (e.g. AL trials similar to PL trials and AR to PR trials in Fig. 2G). At the go-cue their activity became similar for trials requiring the same action (e.g. AL trials became similar to PR trials), and the same was true for the units in the *Q*-value layer.

In our final experiment with this task, we investigated if working memories are formed specifically for task-relevant features. We used the same stimuli, but we now only required pro-saccades so that the color of the fixation point became irrelevant. We trained 100 networks, of which 96 learned the task and we investigated the similarities of the activation patterns. In these networks, the memory units became tuned to cue-location but not to color of the fixation point (Fig. 2H; note the similar activity patterns for trials with a differently colored fixation point, e.g. AL and PL trials). Thus, AuGMEnt specifically induces selectivity for task-relevant features in the association layer.

### Delayed match-to-category task

**Fig 3:**
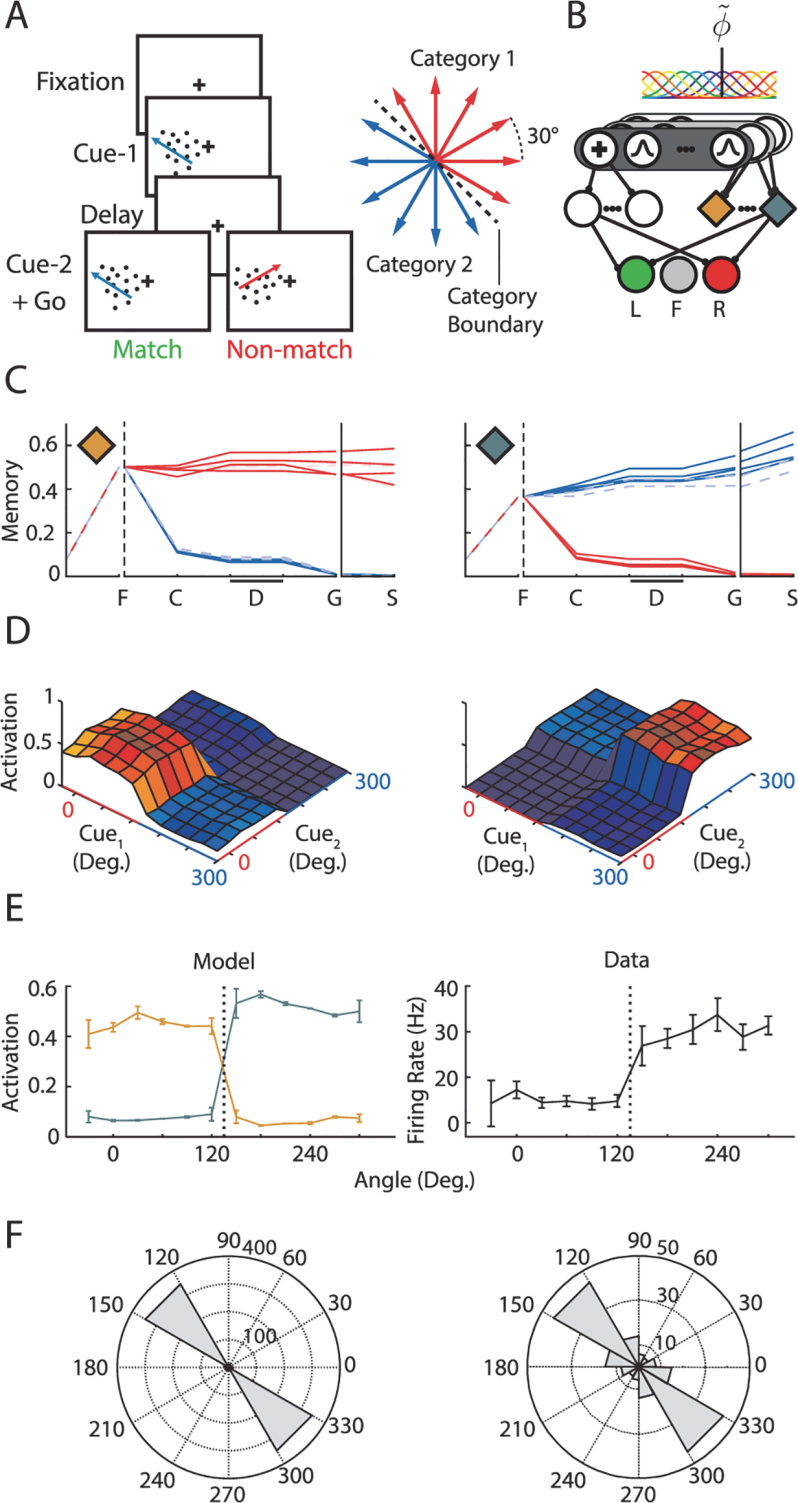
Match-to-category task. ***A***, When the network directed gaze to the fixation point, we presented a motion stimulus (cue-1), and after a delay a second motion stimulus (cue-2). The network had to make a saccade to the left when the two stimuli belonged to the same category (match) and to the right otherwise. There were twelve motion directions, which were divided into two categories (right). ***B***, The sensory layer had a unit representing the fixation point and 20 units with circular Gaussian tuning curves (s.d. 12 deg.) with preferred directions evenly distributed over the unit circle. ***C***, Activity of two example memory units in a trained network evoked by the twelve cue-1 directions. Each line represents one trial, and color represents cue category. Responses to cues closest to the categorization boundary are drawn with a dashed line of lighter color. F, fixation mark onset; C, cue-1 presentation. D, delay; G, cue-2 presentation (go signal); S, saccade. ***D***, Activity of the same two example memory units as in (C) in the ‘go’ phase of the task for all 12x12 combinations of cues. Colors of labels and axes indicate cue category. ***E***, Left, Motion tuning of the memory units (in C) at the end of the memory delay. Error bars show s.d. across trials and the dotted vertical line indicates the category boundary. Right, Tuning of a typical LIP neuron (from [4]), error bars show s.e.m. ***F***, Left, Distribution of the direction change that evoked the largest difference in response across memory units from 100 networks. Right, Distribution of direction changes that evoked largest response differences in LIP neurons (from [4]).

The selectivity of neurons in the association cortex of monkeys changes if the animals are trained to distinguish between categories of stimuli. After training, neurons in frontal [42] and parietal cortex [4] respond similarly to stimuli from the same category and discriminate between stimuli from different categories. In one study [4], monkeys had to group motion stimuli in two categories in a delayed-match-to-category task (Fig. 3A). They first had to look at a fixation point, then a motion stimulus appeared and after a delay a second motion stimulus was presented. The monkeys’ response depended on whether the two stimuli came from the same category or from different categories. We investigated if AuGMEnT could train a network with an identical architecture (with 3 regular and 4 memory units in the association layer) as the network of the delayed saccade/antisaccade task to perform this categorization task. We used an input layer with a unit for the fixation point and 20 units with circular Gaussian tuning curves of the form $r\left(x\right)=\text{exp}\left(-\frac{{\left(x-\theta_{c}\right)}^{2}}{2\sigma^{2}}\right)$ with preferred directions *θ_c_* evenly distributed over the unit circle and a standard deviation *σ* of 12 deg (Fig. 3B). The two categories were defined by a boundary that separated the twelve motion directions (adjacent motion directions were separated by 30 deg.) into two sets of six directions each.

We first waited until the model directed gaze to the fixation point. Two time-steps after fixation we presented one of twelve motion-cues (cue-1) for one time step and gave the fixation reward *r_i_* (Fig. 3A). We added Gaussian noise to the motion direction (s.d. 5 deg.) to simulate noise in the sensory system. The model had to maintain fixation during the ensuing memory delay that lasted two time steps. We then presented a second motion stimulus (cue-2) and the model had to make an eye-movement (either left or right; the fixation mark did not turn off in this task) that depended on the match between the categories of the cues. We required an eye movement to the left if both stimuli belonged to the same category and to the right otherwise, within eight time-steps after cue-2. We trained 100 models and measured accuracy for the preceding 50 trials with the same cue-1. We determined the duration of the learning phase as the trial where accuracy had reached 80% for all cue-1 types.

In spite of their simple feedforward structure with only seven units in the association layer, AuGMEnT trained the networks to criterion in all simulations within a median of 11,550 trials. Fig. 3C illustrates motion tuning of two example memory neurons in a trained network. Both units had become category selective, from cue onset onwards and throughout the delay period. Fig. 3D shows the activity of these units at ‘Go’ time (i.e. after presentation of cue-2) for all 144 combinations of the two cues. Fig. 3E shows the tuning of the memory units during the delay period. For every memory unit of the simulations (*N* = 400), we determined the direction change eliciting the largest difference in activity (Fig. 3F) and found that the units exhibited the largest changes in activity for differences in the motion direction that crossed a category boundary, as do neurons in LIP [4] (Fig. 3E,F, right). Thus, AuGMEnT can train networks to perform a delayed match-to-category task and it induces memory tuning for those feature variations that matter.

### Probabilistic decision making task

We have shown that AuGMEnT can train a single network to perform a delayed saccade/anti-saccade task or a match-to-category task and to maintain task-relevant information as persisitent activity. Persistent activity in area LIP has also been related to perceptual decision making, because LIP neurons integrate sensory information over time in decision making tasks [43]. Can AuGMEnT train the very same network to integrate evidence for a perceptual decision?

**Fig 4:**
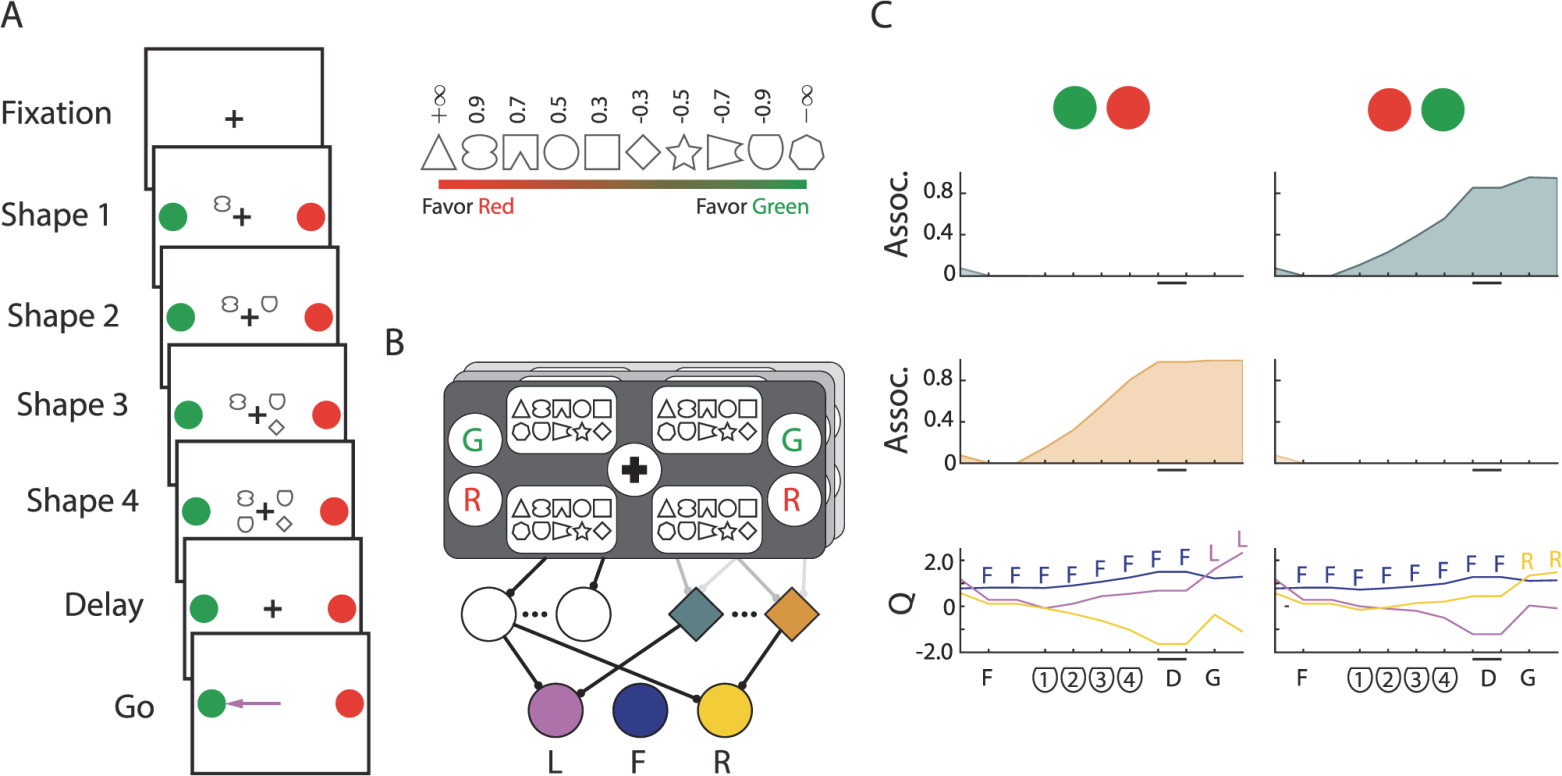
Probabilistic classification task. ***A***, After the network attained fixation, we presented four shapes in a random order at four locations. The shapes *s*_1_,⋯,*s*_4_ cued a saccade to the red or green target: their location varied randomly across trials. Reward was assigned to the red target with probability $P(R|s_{1},s_{2},s_{3},s_{4})=\frac{10^{W}}{1+10^{W}}$, with $W=\sum_{i=1}^{4}w_{i}$, and to the green target otherwise. Inset shows weights *w_i_* associated with cues *s_i_*. ***B***, The sensory layer had units for the fixation point, for the colors of the targets on each side of the screen and there was a set of units for the symbols at each of the four retinotopic locations. ***C***, Activity of two context sensitive memory units and Q-value units (bottom) in a trial where four shield-shaped symbols were presented to a trained network. The green target is the optimal choice. F: fixation mark onset; D: memory delay; G: fixation mark offset (‘Go’-signal).

We focused on a recent study [5] in which monkeys saw a red and a green saccade target and then four symbols that were presented successively. The four symbols provided probabilistic evidence about whether a red or green eye-movement target was baited with reward (Fig. 4A). Some of the symbols provided strong evidence in favor of the red target (e.g. the triangle in the inset of Fig. 4A), others strong evidence for the green target (heptagon) and other symbols provided weaker evidence. The pattern of choices revealed that the monkeys assigned high weights to symbols carrying strong evidence and lower weights to less informative ones. A previous model with only one layer of modifiable synapses could learn a simplified, linear version of this task where the symbols provided direct evidence for one of two actions [44]. This model used a pre-wired memory and it did not simulate the full task where symbols only carry evidence about red and green choices while the position of the red and green targets varied across trials. Here we tested if AuGMEnT could train our network with three regular and four memory units to perform the full non-linear task.

We trained the model with a shaping strategy using a sequence of tasks of increasing complexity, just as in the monkey experiment [5]. We will first decribe the most complex version of the task. In this version, the model (Fig. 4B) had to first direct gaze to the fixation point. After fixating for two time-steps, we gave the fixation reward *r_i_* and presented the colored targets and also one of the 10 symbols at one of four locations around the fixation mark, In the subsequent three time-steps we presented the additional symbols. We randomized location of the red and green targets, the position of the successively presented symbols as well as the symbol sequence over trials. There was a memory delay of two time steps after all symbols (*s*_1_,⋯,*s*_4_) had been presented and we then removed the fixation point, as a cue to make a saccade to one of the colored targets. Reward *r_f_* was assigned to the red target with probability $P(R|s_{1},s_{2},s_{3},s_{4})=\frac{10^{W}}{1+10^{W}}$, with $W=\sum_{i=1}^{4}w_{i}$ (*w_i_* is specified in Fig. 4A, inset) and to the green target otherwise. The model’s choice was considered correct if it selected the target with highest reward probability, or either target if reward probabilities were equal. However, *r_f_* was only given if the model selected the baited target, irrespective of whether it had the highest reward probability.

The shaping strategy used for training gradually increased the set of input symbols (2,4,⋯,10) and sequence length (1,⋯,4) in eight steps (Table 3). Training started with the two `trump' shapes which guarantee reward for the correct decision (triangle and heptagon, see Fig. 4A, inset). We judged that the task had been learned when the success rate in the last *n* trials was 85%. As the number of possible input patterns grew we increased *n* to ensure that a significant fraction of possible input-patterns had been presented before we determined convergence (see Table 3). Difficulty was first increased by adding the pair of symbols with the next smaller absolute weight, until all shapes had been introduced (level 1–5) and then by increasing sequence length (level 6–8).

**Table 3:** Probabilistic Classification convergence windows.

Task difficulty	# Input Symbols	Sequence Length	*n* trials to determine success
**1**	2	1	1,000
**2**	4	1	1,500
**3**	6	1	2,000
**4**	8	1	2,500
**5**	10	1	3,000
**6**	10	2	10,000
**7**	10	3	10,000
**8**	10	4	20,000

With this shaping strategy AuGMEnT successfully trained 99 of 100 networks within a total of 500,000 trials. Training of the model to criterion (85% correct in the final task) took a median total of 55,234 trials across the eight difficulty levels, which is faster than the monkeys learned. After the training procedure, the memory units had learned to integrate information for either the red or green choice over the symbol sequence and maintained information about the value of this choice as persistent activity during the memory delay. Fig. 4C shows the activity of two memory units and the *Q*-value units of an example network during a trial where the shield symbol was presented four times, providing strong evidence that the green target was baited with reward. The memory units became sensitive to the context determined by the position of the red and green saccade targets. The unit in the first row of Fig. 4C integrated evidence for the green target if it appeared on the right side and the unit in the second row if the green target appeared on the left. Furthermore, the activity of these memory units ramped up gradually as more evidence accumulated.

The activity of neurons in LIP was correlated to the log likelihood that the targets are baited [5]. To investigate the influence of log likelihood on the activity of the memory units, we computed log likelihood ratio (logLR) quintiles as follows. We enumerated all 10,000 length 4 symbol combinations *s*∈*S* and computed the probability of reward for a saccade to the red target, *P*(*R*|*S*) for every combination. We next computed the conditional probabilities of reward *P*(*R*|*s_l_*) and *P*(*G*|*s_l_*) = 1-*P*(*R*|*s_l_*) for sequences *s_l_* of length *l*∈{1,⋯,4} (marginalizing over the unobserved symbols). We then computed *LogLR*(*s_l_*) as log_10_(*P*(*R*|*s_l_*)/*P*(*G*|*s_l_*)) for each specific sequence of length *l* and divided those into quintiles.

**Fig 5:**
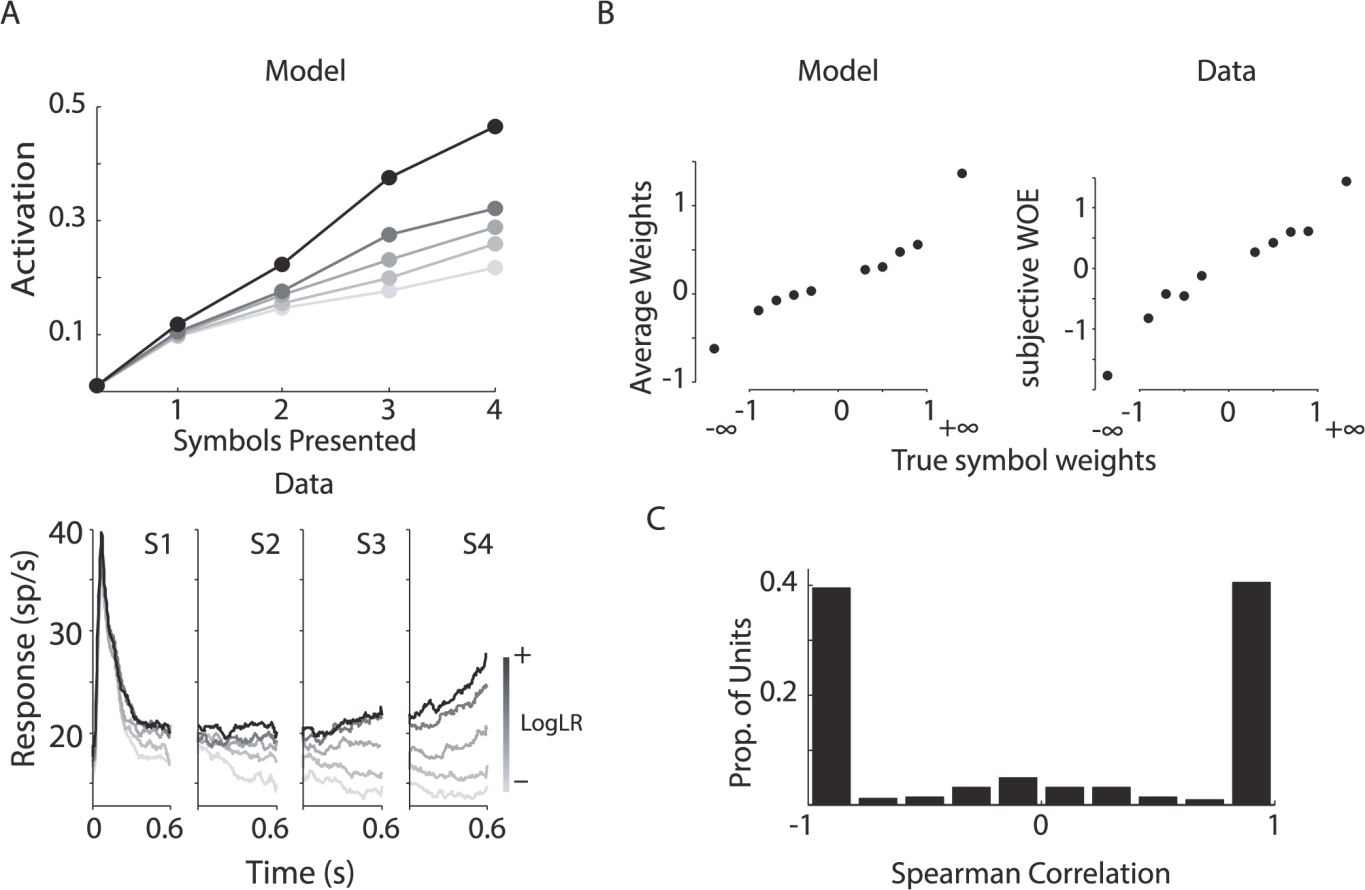
Tuning in the association layer in the probabilistic classification task. ***A***, Trials were subdivided in quintiles based on the log-likelihood ratio of the evidence favoring one target. Average activations of the four memory units of a trained model network (top; 100,000 trials) and LIP neurons (bottom, from [5]) depend on the log-likelihood ratio. ***B***, Left, Average synaptic weights between input units representing symbols and an example memory unit are strongly correlated (*ρ*≈1, *p*<10^-6^) with true symbol weights. Right, Subjective weights assigned by a monkey as estimated from the performance data (from [5]). ***C***, Histogram of Spearman correlations between average synaptic weights for symbols and true symbol weights for 396 memory units (AuGMEnT trained 99 of 100 simulated networks to criterion). Note that there are also units with zero correlation that do not contribute to the mapping of the symbols onto *Q*-values. These units were accompanied by other association units with stronger correlations.

To determine how the activity of memory units depended on the log likelihood that the targets were baited we first compared their average activity after observing a complete sequence of the lower and upper quintile, and reordered the quintiles so they were increasing for each unit. We then computed the average within-quintile activities over the aligned population. The upper panel of Fig. 5A shows how the average activity of the four memory units of an example network depended on the log likelihood that the targets were baited and the lower panel shows LIP data [5] for comparison. It can be seen that the memory units’ activity became correlated to the log likelihood, just like LIP neurons. Importantly, the synaptic weights from input neurons to memory cells depended on the true weights of the symbols after learning (Fig. 5B). This correlation was also strong at the population level as can be seen in Fig. 5C which shows the distribution of all the correlation coefficients (N = 396). Thus, plasticity of synapses onto the memory neurons can explain how the monkeys valuate the symbols and AuGMEnT explains how these neurons learn to integrate the most relevant information. Furthermore, our results illustrate that AuGMEnT not only trains the association units to integrate stochastic sensory evidence but that it also endows them with the required mixed selectivity for target color and symbol sequence that is required to solve this non-linear task [41].

### Vibrotactile discrimination task

**Fig 6:**
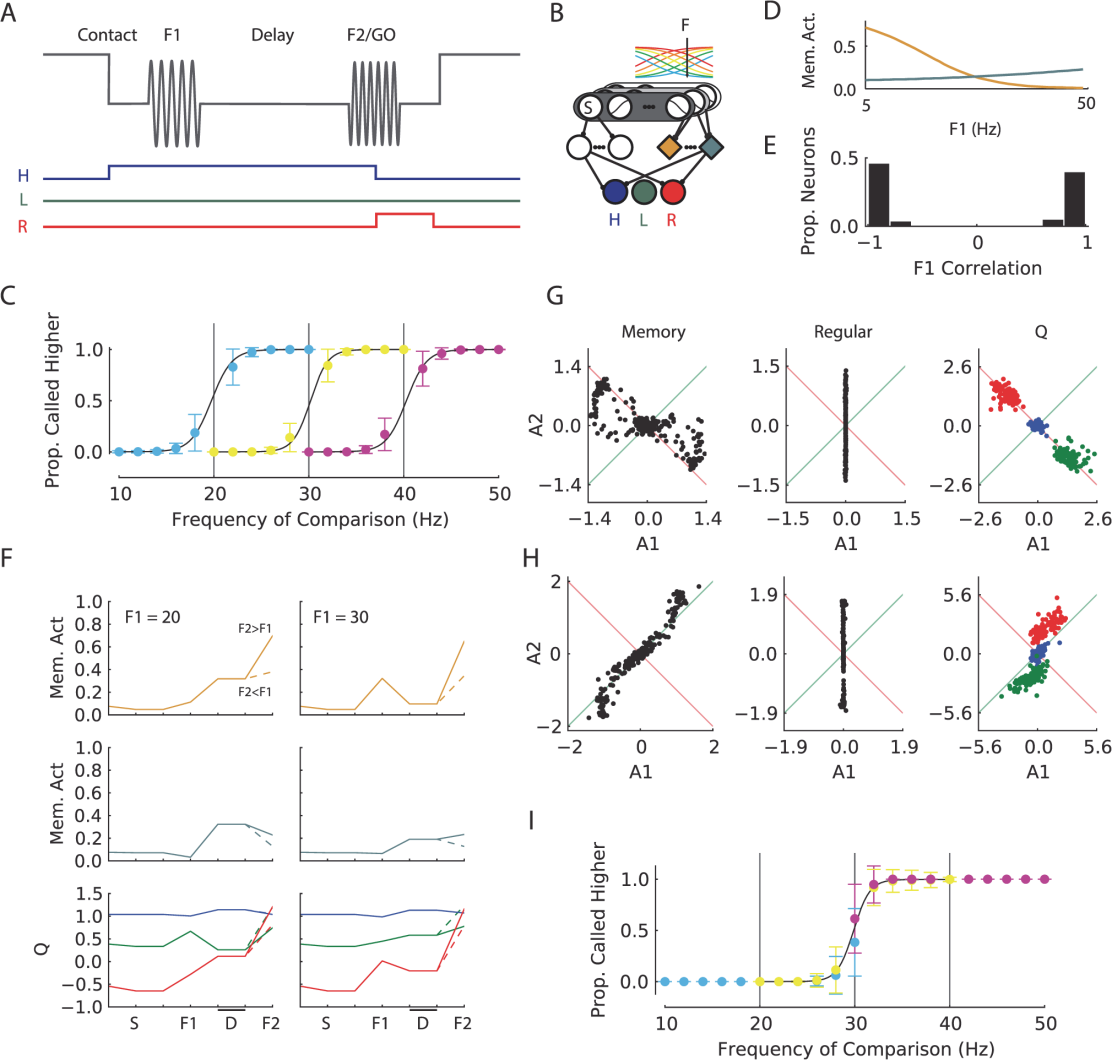
Vibrotactile discrimination task. ***A***, Top line shows vibrotactile stimuli, bottom colored lines show target actions for the example trial (F1 < F2). H, hold key; L, press left button to indicate F2 < F1; R, press right button to indicate F2 > F1. ***B***, Network model. The units in the sensory layer are tuned for the tactile frequency with monotonically increasing or decreasing sigmoidal tuning curves. The binary ‘S’ neuron codes for skin contact of the vibrotactile probe and becomes active at ‘Contact’ in A. ***C***, Average psychometric curves for 100 networks trained on the variable F1 task. Each set of data points (grouped by color) shows responses for the F1 stimulus that is indicated with a vertical line for flanking F2 stimuli; blue: F1 = 20Hz, yellow: F1 = 30Hz and pink: F1 = 40Hz. Y-axis shows the mean proportion of trials where networks indicated that F2 > F1 (each comparison was evaluated 100 times for every network). Error bars show s.d. over networks. Curves are logistic fits to the model responses. ***D***, Tuning of two example memory units to F1 frequency during the delay phase. ***E***, Histogram of linear correlations between F1 frequency and memory unit activations during the delay phase for 100 networks (N = 400). ***F***, Example activity traces for two memory units and the three Q-value units. Left panel shows the response for F1 = 20Hz and F2 = F1±5Hz (solid +5Hz, dashed -5Hz). The response of the *Q*-value units is coded following the color scheme in panels A and B. Right panel shows activity of these units when F1 was 30 Hz. F2 indicates onset of second vibration stimulus. D: Memory delay phase. Note that F2 is 25Hz for the continuous lines in the left panel and also for the dashed lines in the right panel, but that these trials require different responses (right button if F1 = 20Hz and left button if F1 = 30Hz). ***G***, Scatter plot of linear regression parameters of various unit types when F2 was presented (as explained in the main text). A positive A1 (A2) parameter indicates that a unit becomes more active for higher F1 (F2). Green line shows y = x and the activity of units on this line is related to the sum of F1 and F2. The red line represents y = -x, and the activity of units on this line represents the difference between F1 and F2. The color scheme for the Q-value units is the same as in (A) and (B). ***H***, Scatter plot of linear regression parameters at the time of F2 presentation for networks trained on the version of the task with fixed F1. ***I***, Psychometric curves for block-trained fixed F1 networks (see main text). Same conventions as for (C). Only the logistic fit (black line) for F1 = 30 Hz is drawn.

The previous simulations addressed tasks that have been employed for the study of neurons in area LIP of monkeys. Our last simulation investigated a task that has been used to study vibrotactile working memory [6,45]. In this task, the monkey touches a key with one hand and then two vibration stimuli are applied sequentially to a fingertip of the other hand (Fig. 6A). The monkey has to indicate whether the frequency of the first vibration stimulus (F1) is higher or lower than the frequency of the second one (F2). At the end of the trial the animal indicates its choice by releasing the key and pressing one of two buttons. The overall structure of the task is similar to that of the visual tasks described above, but the feature of interest here is that it requires a comparison between two scalar values; F2 that is sensed on the finger and F1 that has to be maintained in working memory.

Recent computational work has addressed various aspects of the vibrotactile discrimination task. Several models addressed how neural network models can store F1 and compare it to F2 [46–48]. More recently, Barak et al. [49] investigated the dynamics of the memory states in networks trained with three different supervised learning methods and compared them to the neuronal data. However, these previous studies did not yet address trial-and-error learning of the vibrotactile discrimination task with a biologically plausible learning rule. We therefore investigated if AuGMEnT could train the same network that had been used for LIP, with three regular units and four memory units, to solve this task.

The input layer was modeled after sensory area S2 of the monkey. Neurons in this cortical area have broad tuning curves and either monotonically increase or decrease their firing rate as function of the frequency of the vibrotactile stimulus [50]. The input units of the model had sigmoidal tuning curves *r*(*x*) = 1/(1+exp(*w*(*θ*_c_-*x*))), with 10 center points *θ*_c_ evenly distributed over the interval between 5.5Hz and 49.5Hz. We used a pair of units at every *θ*_c_ with one unit increasing its activity with stimulus frequency and the other one decreasing, so that there were a total of 20 input units. Parameter *w* determines the steepness of the tuning curve and was +/- 5. We modeled sensory noise by adding independent zero mean Gaussian noise (s.d. 7.5%) to the firing rates of the input units. We also included a binary input unit that signaled skin contact with the stimulation device (unit S in Fig. 6B). The association and *Q*-value layers were identical to those of the other simulations (Fig. 6B).

Our first simulation addressed a version of the task where F1 varied from trial to trial [6]. A trial started when the input unit indicating skin contact with the vibrating probe became active and the model had to select the hold-key within ten time-steps, or else the trial was terminated. When the model had held the key for two time-steps, a vibration stimulus (F1, uniformly random between 5 and 50 Hz) was presented to the network for one time-step and the small shaping reward (*r_i_*) was given. This was followed by a memory delay after which we presented the second vibration stimulus (F2), drawn from a uniform distribution between 5 and 50 Hz, but with a minimal separation of 2 Hz from F1. If F2 was lower than F1 the model had to select the left button (green *Q*-value unit in Fig. 6B)—and the right button (red) otherwise—within eight time steps after the presentation of F2 to obtain the reward *r_f_*.

To determine model performance, we divided the range of F1 stimuli into 9 bins of 5 Hz and kept track of the running average of performance in 50 trials for each bin. When the model reached a performance of 80% for every F1 we disabled learning and exploration (setting learning parameters *β* and *ε* to zero) and checked the performance of the model for F1 stimuli of 20, 30 and 40 Hz and F2 stimuli with offsets of [-10, -8, …, -2,2, …, 8, 10] Hz, repeating each test 20 times. We considered learning to be successful if the model classified the nearest F2 frequencies (2 Hz distance) with a minimal accuracy of 50% and all other F2 frequencies with an accuracy better than 75%, for every F1 bin.

**Fig 7:**
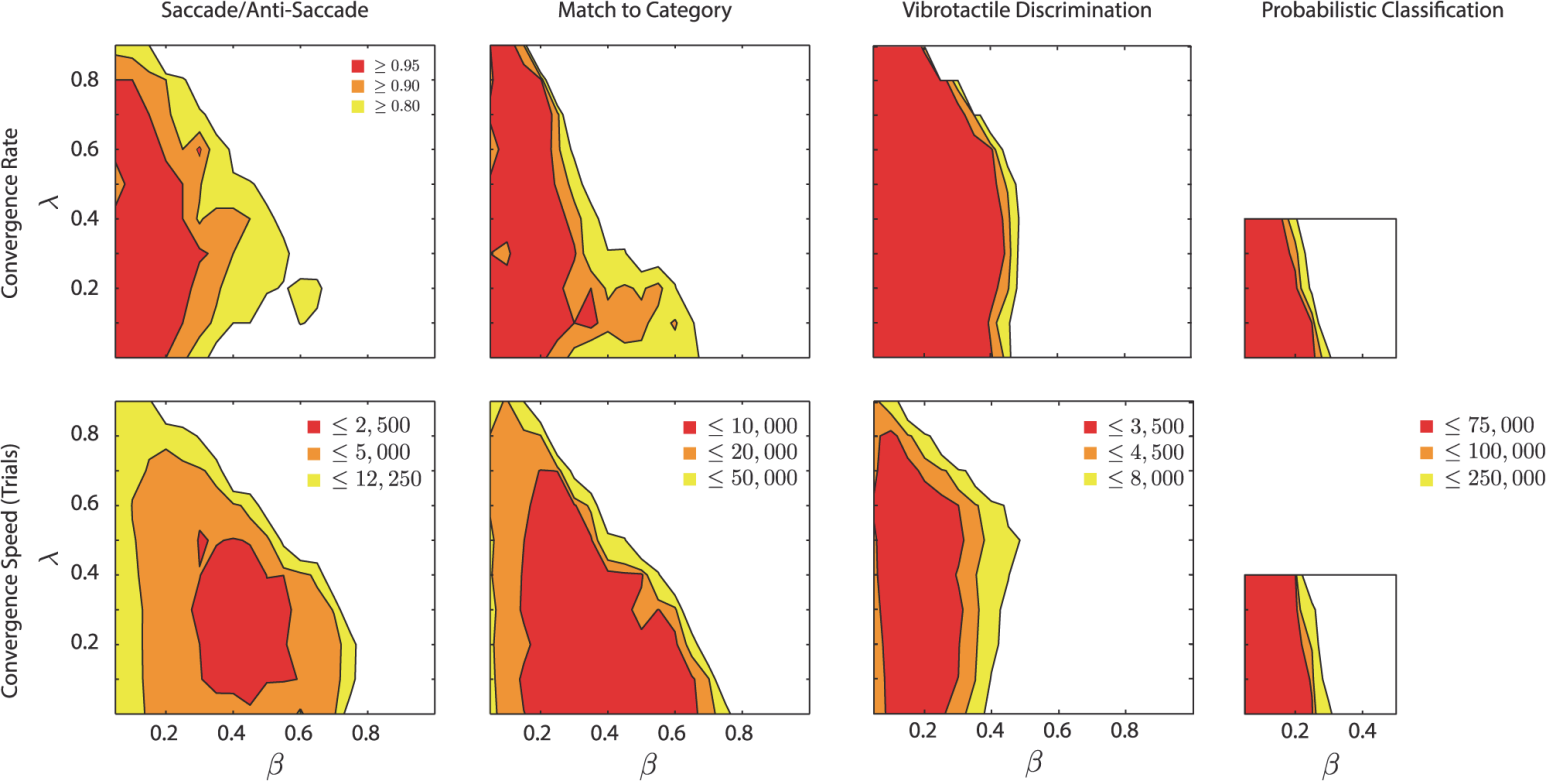
Robustness to variations in the parameters that control learning rate. The upper row shows how the proportion of networks that converged varies as function of *β* (learning rate) and *λ* (decay of tags); white regions had a proportion of convergence lower than 0.8. The lower row shows the effect of *β* and *λ* on the median trial when the learning criterion was reached; white regions reached convergence later than the yellow regions (see insets).

AuGMEnT trained all 100 simulated networks to criterion within a median of 3,036 trials. Fig. 7C illustrates the average (±s.d.) choices of these 100 trained models as a function of F2, for three values of F1 as well as a logistic function fitted to the data [as in 6]. It can be seen that the model correctly indicates whether F1 is higher or lower than F2 and that the criterion depends on the value F1, implying that the model has learned to store this analog scalar value in its working memory. What are the memory representations that emerged during learning? Fig. 6D shows the F1 tuning of two memory units in an example network; typically the tunings are broad and can be increasing or decreasing as a function of F1, similar to what was found in experiments in the frontal cortex of monkeys [51]. Fig. 6E shows the distribution of linear correlations between 400 memory units in 100 trained networks and F1 frequency; most units exhibit a strong positive or negative correlation, indicating that the networks learned to code the memory of F1 as the level of persistent firing of the memory units.

We next investigated how the model carried out the comparison process that has to take place after the presentation of F2. This comparison process depends critically on the order of presentation of the two stimuli, yet it involves information that comes in via the same sensory inputs and association units [48]. We found that the memory units were indeed sensitive to both F1 and F2 in the comparison period. Fig. 6F shows the response of two example memory units and the three *Q*-value units for a trials with an F1 of 20 or 30 Hz, followed by an F2 with a frequency that was either 5Hz higher (solid line) or lower than F1 (dashed line). The activity of the memory units encodes F1 during the memory delay, but these units also respond to F2 so that the activity after the presentation of F2 depends on both frequencies. The lower panel illustrates the activity of the *Q*-value units. The activity of the Hold *Q*-value unit (H, blue) is highest until the presentation of F2, causing the model to hold the key until the go-signal. This unit did not distinguish between trials that required a right or left button press. The activities of *Q*-value units for the left and right button press (red and green traces) explain how the network made correct decisions at the go-signal because the *Q*-value of the appropriate action became highest (the solid lines in Fig. 6F show activity if F2>F1 and dashed lines F2<F1). It can be seen, for example, how the response elicited in the *Q*-value layer by an F2 of 25Hz depended on whether the preceding F1 was 20Hz (continuous curves in the left panel of Fig. 6F) or 30Hz (dashed curves in the right panel).

We next quantified how the activity of the memory, regular and *Q*-value units from 100 networks (*N* = 400, 300 and 300 units, respectively) depended on F1 and F2 during the comparison phase with a regression [see 52] using all trials where the F2 stimulus was presented and for all combinations of the two frequencies between 5 and 50 Hz (step size 1Hz),

$$r(F1,F2)=F1a_{1}+F2a_{2}+b$$ (32)

Here *a_1_* and *a_2_* estimate the dependence of the unit’s activity on F1 and F2, respectively. The activity of many memory units depended on F1 and also on F2 (Fig. 6G, left) and the overall negative correlation between the coefficients (*r* = -0.81, *p*<10^-6^) indicates that units that tended to respond more strongly for increasing F1 tended to decrease their response for increasing F2 and vice versa, just as is observed in area S2, the prefrontal cortex and the medial premotor cortex of monkeys [45,51,52]. In other words, many memory units became tuned to the difference between F1 and F2 in the comparison phase, as is required by this task. In spite of the fact that F1 and F2 activate memory units with the same synapses, the inverse tuning is possible because the F1 stimulus has turned off and activated the off-cells in the sensory layer in the comparison phase. In contrast, the F2 stimulus is still ‘on’ in this phase of the task so that the off-units coding F2 did not yet provide their input to the memory cells. As a result, the memory units’ final activity can reflect the difference between F1 and F2, as is required by the task. Regular units only have access to the current stimulus, and were therefore they are only tuned to F2 in the comparison phase (Fig. 6G, middle). *Q*-value units reflect the outcome of the comparison process (Fig. 6G, right): their regression coefficients with F1 and F2 fall into three clusters as predicted by the required action.

The version of the task described above demanded the comparison between two flutter frequencies because F1 varied from trial to trial. Hernández et al. [6] also studied a version of the task where F1 was fixed for a block of trials. In this version, the monkeys based their response on F2 only and did not memorize F1. As a result their performance deteriorated at the start of a new block of trials with a different F1. Networks trained with AuGMEnT also only memorize task-relevant information. Do networks trained with AuGMEnT also fail to memorize F1 if it is fixed during training? To investigate this question, we trained models with a fixed F1 of 30 Hz [6] and presented F2 stimuli in the range between 5–50 Hz (2.5 Hz spacing) with a minimal distance from F1 of 10 Hz. We estimated convergence as the trial when accuracy reached 90% (running average of 50 trials).

AuGMEnT trained all 100 networks to criterion in this simpler task within a median of 1,390 trials. After learning the fixed F1 task, we subjected the networks to block training with F1 stimuli of 20, 30 and 40 Hz as in [6] while we presented F2 stimuli with frequencies of ([-10,-8, …,-2,2,…, 8,10] Hz relative to F1 (10 total, each shown 150 times). These blocks of trials had a pseudorandom ordering but we always presented a 30Hz F1 in the last block. When we tested immediately after every block, we found that the models were well able to adapt to a specific F1. However, the models were not able to solve the variable F1 task after this extensive block training, even though they had significant exposure to different F1 stimuli. Fig. 6I shows the average psychometric curves for 100 networks after the last block with F1 = 30Hz. Colors represent trials with different F1 stimuli (as in Fig. 6C). It can be seen that the models disregarded F1 and only determined whether F2 was higher or lower than 30 Hz, just as monkeys that are trained with a blocked procedure [6]. Thus, the model can explain why the monkeys do not learn to compare the two stimuli if the F1 is fixed for longer blocks of trials. The memory units and the *Q*-value units now had similar rather than opposite tuning for F1 and F2 (positive correlations in the left and right panel of Fig. 6H; compare to Fig. 6G), which indicates that blocked training causes a failure to learn to subtract the memory trace of F1 from the representation of F2.

We conclude that AuGMEnT is able to train networks on a task that requires a comparison between two analog stimuli and where the correct decision depends on stimulus order. Memory units learn to represent the analog value that needs to be memorized as a graded level of persistent activity. However, if F1 is fixed for blocks of trials, the network does not memorize F1 but learns to base its decision on F2 only, in accordance with experimental findings.

### Varying the learning parameters and the size of the network

It is remarkable that AuGMEnT can train the same simple network to perform a wide range of tasks, simply by delivering rewards at the appropriate times. In the simulations described above we fixed the number of units in the association layer and *Q*-value layer and used a single set of learning parameters. To examine the stability of the learning scheme, we also evaluated learning speed and convergence rate for various values of the learning rate *β* and the SARSA learning parameter *λ* (which determines the tag-decay parameter *α* because *α* = 1-*λγ* as was explained above, *γ* was kept at the default value). For the saccade/antisaccade, match-to-category and vibrotactile discrimination tasks we tested *β*∈{0.05,0.10,⋯,1.0} and λ∈{0.0,0.1,⋯,0.9} while the other parameters remained the same (Table 1,2) and ran 100 simulations for every combination. Fig. 7 shows the proportion of networks that converged and the median convergence trial. Training in the probabilistic classification task required a number of different training stages and a longer overall training time and we evaluated this task with a smaller set of parameters (Fig. 7, right). There was a wide range for the learning parameters where most of the networks converged and these ranges overlapped for the four tasks, implying that the AuGMEnT learning scheme is relatively robust and stable.

**Fig 8:**
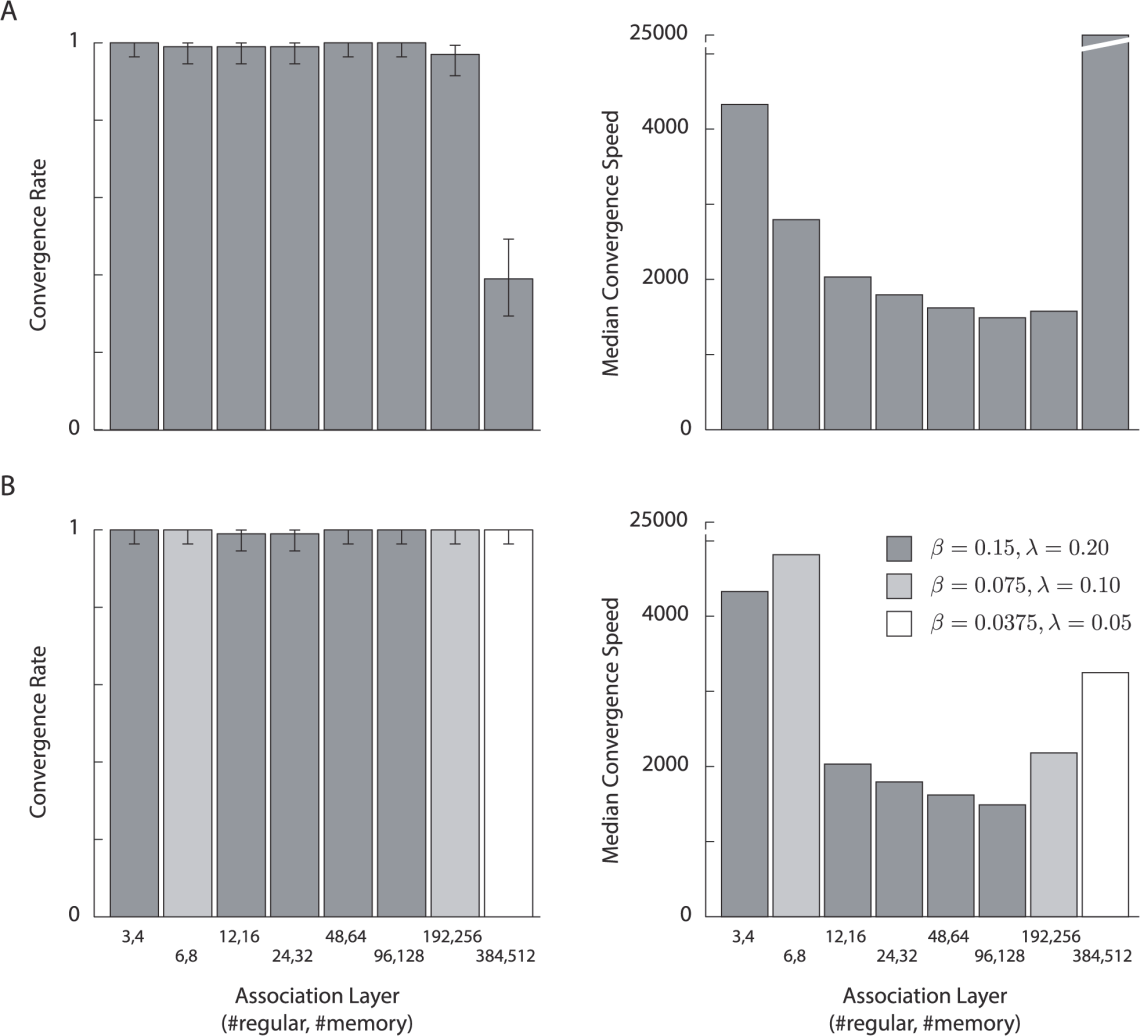
Varying the size of the association layer. ***A***, Scaling with unchanged learning parameters *β* and *λ*. Left, convergence rate (proportion of 100 networks that learned the saccade/antisaccade task). Error bars denote 95% confidence intervals. Right, median convergence speed (number of trials to criterion). ***B***, Left, convergence rates with adjusted learning parameters. Bar shading indicates parameter setting (see legend in right panel). Right, median convergence speed with optimized parameters.

So far our simulations used a fixed network with only 7 units in the association layers. Can AuGMEnT also train networks with a larger association layer? To further investigate the generality of the learning scheme, we ran a series of simulations with increasing numbers of association units, multiplying the number of association units in the network described above by 2, 4, …, 128 and training 100 networks of each size in the saccade/antisaccade task. We first evaluated these larger networks without changing the learning parameters and found that the learning was largely unaffected within a limited range of network sizes, whereas performance deteriorated for networks that were 32–128 fold larger (Fig. 8A). The decrease in performance is likely caused by the larger number of synapses, causing larger adjustments of the *Q*-values after each time step than in the smaller networks. It is possible to compensate for this effect by choosing a smaller *β* (learning rate) and *λ*. We jointly scaled these parameters by $\frac{1}{2},\frac{1}{4}$ and $\frac{1}{8}$ and selected the parameter combination which resulted in the highest convergence rate and the fastest median convergence speed for every network size (Fig. 8B). The performance of the larger networks was at least as good as that of the network with 7 units if learning parameters were scaled. Thus, AuGMEnT can also successfully train networks with a much larger association layer.

## Discussion

AuGMEnT provides a new theoretical framework that can explain how neurons become tuned to relevant sensory stimuli in sequential decision tasks during trial-and-error learning. The scheme uses units inspired by transient and sustained neurons in sensory cortices [19], action-value coding neurons in frontal cortex, basal ganglia and midbrain [12,35,36] and neurons with mnemonic activity that integrate input in association cortex. To the best of our knowledge, AuGMEnT is the first biologically plausible learning scheme that implements SARSA in a multi-layer neural network equipped with working memory. The model is simple, yet is able to learn a wide range of difficult tasks requiring non-linear sensory-motor transformations, decision making, categorization, and working memory. AuGMEnT can train the very same network to perform either of these tasks by presenting the appropriate sensory inputs and reward contingency, and the representations it learns are similar to those found in animals trained on these tasks. AuGMEnT is a so-called on-policy method because it only relies on the *Q*-values that the network experiences during learning. These on-policy methods appear to be more stable than off-policy algorithms (such as *Q*-learning which considers transitions not experienced by the network), if combined with neural networks (see e.g. [53,54]).

AuGMEnT forms memory representations for features that need to be remembered. In the delayed saccade/anti-saccade task, training induced persistent neuronal activity tuned to the cue location and to the color of the fixation point, but only if it was relevant. In the categorization task, units became sensitive to category boundaries and in the decision making task, units integrated sensory evidence with stronger weights for the more reliable inputs. These properties resemble those of neurons in LIP [2–5] and the frontal cortex [24] of monkeys. Finally, the memory units learned to memorize and compare analog values in the vibrotactile task, just as has been observed in the frontal cortex of monkeys [6,45].

AuGMEnT makes a number of predictions that could be tested in future neuroscientific experiments. The first and foremost prediction is that feedback connections gate plasticity of the connections by inducing synaptic tags. Specifically, the learning scheme predicts that feedback connections are important for the induction of tags on feedforward connections from sensory cortices to the association cortex (Fig. 1B). A second prediction is the existence of traces in synapses onto neurons with persistent activity (i.e. memory units) that are transformed into tags upon the arrival of feedback from the response selection stage, which may occur at a later point in time. The third prediction is that these tags interact with globally released neuromodulators (e.g. dopamine, acetylcholine or serotonin), which determine the strength and sign of the synaptic changes (potentiation or depression). Neurobiological evidence for the existence of these tags and their interaction with neuromodulatory substances will be discussed below. A final prediction is that stationary stimuli provide transient input to neurons with persistent activity. As a result, stimuli that are visible for a longer time do not necessarily cause a ramping of activity. In our network ramping was prevented because memory units received input from “on” and “off” input units only. We note, however, that other mechanisms such as, for example, rapidly adapting synapses onto memory cells, could achieve the same effect. In contrast, neurons in association cortex without persistent activity are predicted to receive continuous input, for as long as a stimulus is present. These specific predictions could all be tested in future neuroscientific work.

### Role of attentional feedback and neuromodulators in learning

AuGMEnT implements a four-factor learning rule. The first two factors are pre- and post-synaptic activity of the units and there are two additional “gating factors” that enable synaptic plasticity. The first gating factor is the feedback from units in the motor layer that code the selected action. These units send an attentional signal back to earlier processing levels to tag synapses responsible for selecting this action. The importance of selective attention for learning is supported by experiments in cognitive psychology. If observers select a stimulus for an action, attention invariably shifts to this stimulus [55] and this selective attention signal gates perceptual learning so that attended objects have larger impact on future behavior [56–58]. Moreover, neurophysiological studies demonstrated that such a feedback signal exists, because neurons in the motor cortex that code an action enhance the activity of upstream neurons providing input for this action [59,60].

The second gating-factor that enables plasticity is a global neuromodulatory signal that broadcasts the RPE to many brain regions and determines the sign and strength of the changes in synapses that have been tagged. Dopamine is often implicated because it is released if reward expectancy increases and it influences synaptic plasticity [10,38]. There is also a potential role for acetylcholine because cholinergic cells project diffusely to cortex, respond to rewards [61–63] and influence synaptic plasticity [61,64]. Furthermore, a recent study demonstrated that serotonergic neurons also carry a reward-predicting signal and that the optogenetic activation of serotonergic neurons acts as a positive reinforcer [65]. Guidance of synaptic plasticity by the combination of neuromodulatory signals and cortico-cortical feedback connections is biologically plausible because all information for the synaptic update is available at the synapse.

### Synaptic tags and synaptic traces

Learning in AuGMEnT depends on synaptic tags and traces. The first step in the plasticity of a synapse onto a memory cell is the formation of a synaptic trace that persists until the end of the trial (Fig. 1C). The second step is the conversion of the trace into a tag, when a selected motor unit feeds back to the memory cell. The final step is the release of the neuromodulator that modifies tagged synapses. The learning rule for the synapses onto the regular (i.e. non-memory) association units is similar (Fig. 1B), but tags form directly onto active synapses, skipping the first step. We note, however, that the same learning rule is obtained if these synapses also have traces that decay within one time-step. The hypothesis that synaptic plasticity requires a sequence of events [66,67] is supported by the synapses’ complex biochemical machinery. There is evidence for synaptic tags [15,31,32] and recent studies have started to elucidate their identity [32]. Neuromodulatory signals influence synaptic plasticity even if released seconds or minutes later than the plasticity-inducing event [15,17,32], which supports the hypothesis that they interact with some form of tag.

### Comparison to previous modeling approaches

There has been substantial progress in biologically inspired reinforcement learning models with spiking neurons [68–71] and with models that approximate population activity with continuous variables [14,16,21,44,67,72–74]. Many of the models rely either on Actor-Critic learning [7] or on policy gradient learning [75]. An advantage of Actor-Critic models is that model components relate to brain regions [16,71,73]. AuGMEnT has features in common with these models. For example, it uses the change in *Q*-value to compute the RPE (Eqn. (17)). Another widely used class of models is formed by policy gradient learning methods [68,75] where units (or synapses [68]) act as local agents that try to increase the global reward. An advantage of these models is that learning does not require knowledge about the influence of units on other units in the network, but a disadvantage is that the learning process does not scale well to larger networks where the correlation between local activity and the global reward is weak [70]. AuGMEnT uses ‘attentional’ feedback from the selected action to improve leaning [14] and it also generalizes to multi-layer networks. It thereby alleviates a limitation of many previous biologically plausible RL models, which can only train a single layer of modifiable synaptic weights and solve linear tasks [16,21,44,67,70,71,73,76] and binary decisions [21,44,67,70].

Unlike these previous models, AuGMEnT is a model of action-value learning (SARSA(*λ*) [7]). It differs from many previous models in its ability to train task-relevant working memory representations, without pre-wiring. We modeled memory units as integrators, because neurons that act as integrators and maintain their activity during memory delays have been found in many cortical regions [2–5,23,24]. To keep the model simple, we did not specify the mechanisms causing persistent activity, which could derive from intracellular processes, local circuit reverberations or recurrent activity in larger networks spanning cortex, thalamus and basal ganglia [20–22].

A few studies included a pre-wired working memory in RL [21,44] but there has been comparatively little work on biologically plausible learning of new memories. Earlier neural networks models used “backpropagation-through-time”, but its mechanisms are biologically implausible [77]. The long short-term memory model (LSTM) [78] is a more recent and popular approach. Working memories in LSTM rely on the persistent activity of memory units, which resemble the ones used by AuGMEnT. However, LSTM relies on the biologically implausible error-backpropagation rule. To our knowledge, only one previous model addressed the creation of working memories with a neurobiologically inspired learning scheme, the prefrontal basal-ganglia working memory model (PBWM) [72], which is part of the Leabra cognitive architecture [79,80]. Although a detailed comparison of AuGMEnT and Leabra is beyond the scope of this article, it is useful to mention a few key differences. First, the complexity and level of detail of the Leabra/PBWM framework is greater than that of AuGMEnT. The PBWM framework uses more than ten modules, each with its own dynamics and learning rules, making formal analysis difficult. We chose to keep the models trained with AuGMEnT as simple as possible, so that learning is easier to understand. AuGMEnT’s simplicity comes at a cost because many functions remained abstract (see next section). Second, the PBWM model uses a teacher that informs the model about the correct decision, i.e. it uses more information than just reward feedback. Third, PBWM is an actor-critic architecture that learns the value of states, whereas AuGMEnT learns the value of actions. Fourth and finally, there are important differences in the mechanisms for working memory. In PBMW, memory units are bi-stable and the model is equipped with a system to gate information in prefrontal cortex via the basal ganglia. In AuGMEnT, memory units are directly activated by on- and off-units in the input layer and they have continuous activity levels. The activity profile of memory units is task-dependent in AuGMEnT. It can train memory units to integrate evidence for probabilistic decision making, to memorize analog values as graded levels of persistent activity but also to store categories with almost binary responses in a delayed match-to-category task.

### Biological plausibility, biological detail and future work

We suggested that AuGMEnT is biologically plausible, but what do we mean with this statement? Our aim was to propose a learning rule based on Hebbian plasticity that is gated by two factors known to gate plasticity: a neuromodulatory signal that is released globally and codes the reward-prediction error and an attentional feedback signal that highlights the part of the network that is accountable for the outcome of an action. We showed that the combination of these two factors, which are indeed available at the level of the individual synapses, can cause changes in synaptic strength that follow gradient descent on the reward-prediction error for the transitions that the network experiences. At the same time, the present model provides only a limited degree of detail. The advantage of such a more abstract model is that it remains mathematically tractable. The downside is that more work will be needed to map the proposed mechanisms onto specific brain structures. We pointed out the correspondence between the tuning that developed in the association layer and tuning in the association cortex of monkeys. We now list a number of simplifying assumptions that we made and that will need to be alleviated by future models that incorporate more biological detail.

First, we assumed that the brain can compute the SARSA temporal difference error, which implies a comparison between the *Q*-value of one state-action combination to the *Q*-value of the next combination. Future modeling studies could include brain structures for storing the *Q*-value of the previously selected action while new action-values are computed. Although we do not know the set of brain structures that store action values, previous studies implicated the medial and lateral prefrontal cortex in storing the outcome that is associated with an action [81,82]. Prefrontal neurons even update the predicted outcome as new information comes in during the trial [83]. An alternative to storing *Q*-values is provided by Actor-Critic architectures that assign values to the various states instead of state-action combinations. They use one network to estimate state-values and another network to select actions [16]. Interestingly, [16] proposed that the basal ganglia could compute temporal difference errors by comparing activity in the indirect pathway, which might store the predicted value of the previous time-step, and the direct pathway, which could code the predicted value of the next state. We hypothesize that a similar circuit could be used to compute SARSA temporal difference errors. In addition, we also did not model the action-selection process itself, which has been suggested to take place in the basal ganglia (see [30]).

A second simplification is that we did not constrain model units to be either inhibitory or excitatory—outgoing weights could have either sign and they could even change sign during learning. Future studies could specify more detailed network architectures with constrained weights ([e.g. as in 72]). Indeed, it is possible to change networks into functionally equivalent ones with excitatory and inhibitory units that have only positive weights [84], but the necessary generalization of AuGMEnT-like learning rules would require additional work.

The third major simplification is that feedback connections in AuGMEnT influence the formation of synaptic tags, but do not influence the activity of units at earlier processing levels. Future studies could include feedback connections that also influence activity of units in the lower layers and develop learning rules for the plasticity of activity propagating feedback connections. These connections might further expand the set of tasks that neural networks can master if trained by trial-and-error. In this context it is of interest that previous studies demonstrated that feedforward propagation of activity to higher cortical areas mainly utilizes the AMPA receptor, whereas feedback effects rely more on the NMDA receptor [85], which plays an important role in synaptic plasticity. NMDA receptors also modify neuronal activity in lower areas, and another candidate receptor that could have a specific role in the influence of feedback connections on plasticity are metabotropic glutamate receptors, which are prominent in feedback pathways [86,87] and known to influence synaptic plasticity [88].

A fourth simplification is that we modeled time in discrete steps and used units with scalar activity levels and differentiable activation functions. Therefore, implementations of AuGMEnT using populations of spiking neurons in continuous time deserve to be studied. We leave the integration of the necessary biological detail in AuGMEnT-like networks that would alleviate all these simplifications for future work.

### Conclusions

Here we have shown that interactions between synaptic tags and neuromodulatory signals can explain how neurons in ‘multiple-demand’ association areas acquire mnemonic signals for apparently disparate tasks that require working memory, categorization or decision making. The finding that a single network can be trained by trial and error to perform these diverse tasks implies that these learning problems now fit into a unified reinforcement learning framework.

## References

[1] Duncan J (2010) The multiple-demand (MD) system of the primate brain: mental programs for intelligent behaviour. Trends Cogn Sci 14: 172–179. 10.1016/j.tics.2010.01.004 20171926

[2] Gnadt JW, Andersen RA (1988) Memory related motor planning activity in posterior parietal cortex of macaque. Exp Brain Res 70: 216–220. 3402565 10.1007/BF00271862

[3] Gottlieb J, Goldberg ME (1999) Activity of neurons in the lateral intraparietal area of the monkey during an antisaccade task. Nat Neurosci 2: 906–912. 10491612 10.1038/13209

[4] Freedman DJ, Assad JA (2006) Experience-dependent representation of visual categories in parietal cortex. Nature 443: 85–88. 16936716 10.1038/nature05078

[5] Yang T, Shadlen MN (2007) Probabilistic reasoning by neurons. Nature 447: 1075–1080. 17546027 10.1038/nature05852

[6] Hernández A, Salinas E, García R, Romo R (1997) Discrimination in the sense of flutter: new psychophysical measurements in monkeys. J Neurosci 17: 6391–6400. 9236247 10.1523/JNEUROSCI.17-16-06391.1997 PMC6568331

[7] Sutton RS, Barto AG (1998) Reinforcement Learning: an introduction MIT Press.

[8] Rumelhart DE, Hinton GE, Williams RJ (1986) Learning representations by back-propagating errors. Nature 323: 533–536.

[9] Schultz W (2007) Multiple Dopamine Functions at Different Time Courses. Annu Rev Neurosci 30: 259–288. 17600522 10.1146/annurev.neuro.28.061604.135722

[10] Montague PR, Hyman SE, Cohen JD (2004) Computational roles for dopamine in behavioural control. Nature 431: 760–767. 15483596 10.1038/nature03015

[11] Dayan P, Balleine BW (2002) Reward, Motivation, and Reinforcement Learning. Neuron 38: 285–298. 10.1016/s0896-6273(02)00963-7 12383782

[12] Morris G, Nevet A, Arkadir D, Vaadia E, Bergman H (2006) Midbrain dopamine neurons encode decisions for future action. Nat Neurosci 9: 1057–1063. 16862149 10.1038/nn1743

[13] Todd MT, Niv Y, Cohen JD (2009) Learning to use working memory in partially observable environments through dopaminergic reinforcement. NIPS 21: 1689–1696.

[14] Roelfsema PR, van Ooyen A (2005) Attention-gated reinforcement learning of internal representations for classification. Neural Comp 17: 2176–2214. 10.1162/0899766054615699 16105222

[15] Cassenaer S, Laurent G (2012) Conditional modulation of spike-timing-dependent plasticity for olfactory learning. Nature 482: 47–52. 10.1038/nature10776 22278062

[16] Houk JC, Adams JL, Barto AG (1995) A model of how the basal ganglia generate and use neural signals that predict reinforcement In: Houk JC, Davis JL, Beiser DG, editors. Models of Information Processing in the Basal Ganglia. MIT Press pp. 1–22. 10.1007/s00422-011-0439-5

[17] Yagishita S, Hayashi-Takagi A, Ellis-Davies GCR, Urakubo H, Ishii S, et al (2014) A critical time window for dopamine actions on the structural plasticity of dendritic spines. Science 345: 1616–1620. 10.1126/science.1255514 25258080 PMC4225776

[18] Rombouts JO, Bohte SM, Roelfsema PR (2012) Neurally Plausible Reinforcement Learning of Working Memory Tasks. NIPS 25 pp. 1880–1888.

[19] Nassi JJ, Callaway EM (2009) Parallel processing strategies of the primate visual system. Nat Rev Neurosci 10: 360–372. 10.1038/nrn2619 19352403 PMC2771435

[20] Koulakov AA, Raghavachari S, Kepecs A, Lisman JE (2002) Model for a robust neural integrator. Nat Neurosci 5: 775–782. 12134153 10.1038/nn893

[21] Engel TA, Wang X-J (2011) Same or Different? A Neural Circuit Mechanism of Similarity-Based Pattern Match Decision Making. J Neurosci 31: 6982–6996. 10.1523/JNEUROSCI.6150-10.2011 21562260 PMC3110065

[22] Fransén E, Tahvildari B, Egorov AV, Hasselmo ME, Alonso AA (2006) Mechanism of Graded Persistent Cellular Activity of Entorhinal Cortex Layer V Neurons. Neuron 49: 735–746. 16504948 10.1016/j.neuron.2006.01.036

[23] Egorov AV, Hamam BN, Fransén E, Hasselmo ME, Alonso AA (2002) Graded persistent activity in entorhinal cortex neurons. Nature 420: 173–178. 12432392 10.1038/nature01171

[24] Funahashi S, Bruce CJ, Goldman-Rakic PS (1989) Mnemonic coding of visual space in the monkey's dorsolateral prefrontal cortex. J Neurophys 61: 331–349. 10.1152/jn.1989.61.2.331 2918358

[25] Wiering M, Schmidhuber J (1997) HQ-learning. Adaptive Behavior 6: 219–246.

[26] Humphries MD, Stewart RD, Gurney KN (2006) A Physiologically Plausible Model of Action Selection and Oscillatory Activity in the Basal Ganglia. J Neurosci 26: 12921–12942. 17167083 10.1523/JNEUROSCI.3486-06.2006 PMC6674973

[27] Usher M, McClelland JL (2001) The time course of perceptual choice: the leaky, competing accumulator model. Psychol Rev 108: 550–592. 11488378 10.1037/0033-295x.108.3.550

[28] Gurney KN, Prescott TJ, Redgrave P (2001) A computational model of action selection in the basal ganglia. I. A new functional anatomy. Biol Cybern 84: 401–410. 11417052 10.1007/PL00007984

[29] Stewart TC, Bekolay T, Eliasmith C (2012) Learning to select actions with spiking neurons in the Basal Ganglia. Front Neurosci 6. 10.3389/fnins.2012.00002 PMC3269066 22319465

[30] Lo C-C, Wang X-J (2006) Cortico–basal ganglia circuit mechanism for a decision threshold in reaction time tasks. Nat Neurosci 9: 956–963. 16767089 10.1038/nn1722

[31] Frey U, Morris RGM (1997) Synaptic tagging and long-term potentiation. Nature 385: 533–536. 9020359 10.1038/385533a0

[32] Moncada D, Ballarini F, Martinez MC, Frey JU, Viola H (2011) Identification of transmitter systems and learning tag molecules involved in behavioral tagging during memory formation. Proc Natl Acad Sci USA 108: 12931–12936. 10.1073/pnas.1104495108 21768371 PMC3150922

[33] Mao T, Kusefoglu D, Hooks BM, Huber D, Petreanu L, et al (2011) Long-Range Neuronal Circuits Underlying the Interaction between Sensory and Motor Cortex. Neuron 72: 111–123. 10.1016/j.neuron.2011.07.029 21982373 PMC5047281

[34] Rummery GA, Niranjan M (1994) On-line Q-learning using connectionist systems. Cambridge.

[35] Hikosaka O (2005) Basal Ganglia Orient Eyes to Reward. J Neurophys 95: 567–584. 10.1152/jn.00458.2005 16424448

[36] Samejima K, Ueda Y, Doya K, Kimura M (2005) Representation of Action-Specific Reward Values in the Striatum. Science 310: 1337–1340. 16311337 10.1126/science.1115270

[37] Padoa-Schioppa C, Assad JA (2006) Neurons in the orbitofrontal cortex encode economic value. Nature 441: 223–226. 16633341 10.1038/nature04676 PMC2630027

[38] Schultz W (2002) Getting formal with dopamine and reward. Neuron 36: 241–263. 12383780 10.1016/s0896-6273(02)00967-4

[39] Krueger KA, Dayan P (2009) Flexible shaping: How learning in small steps helps. Cognition 110: 380–394. 10.1016/j.cognition.2008.11.014 19121518

[40] Sommer MA, Wurtz RH (2001) Frontal eye field sends delay activity related to movement, memory, and vision to the superior colliculus. J Neurophys 85: 1673–1685. 10.1152/jn.2001.85.4.1673 11287490

[41] Rigotti M, Barak O, Warden MR, Wang X-J, Daw ND, et al (2013) The importance of mixed selectivity in complex cognitive tasks. Nature 497: 585–590. 10.1038/nature12160 23685452 PMC4412347

[42] Freedman DJ, Riesenhuber M, Poggio T, Miller EK (2001) Categorical representation of visual stimuli in the primate prefrontal cortex. Science 291: 312–316. 11209083 10.1126/science.291.5502.312

[43] Gold JI, Shadlen MN (2007) The Neural Basis of Decision Making. Annu Rev Neurosci 30: 535–574. 17600525 10.1146/annurev.neuro.29.051605.113038

[44] Soltani A, Wang X-J (2009) Synaptic computation underlying probabilistic inference. Nat Neurosci 13: 112–119. 10.1038/nn.2450 20010823 PMC2921378

[45] Romo R, Brody CD, Hernández A, Lemus L (1999) Neuronal correlates of parametric working memory in the prefrontal cortex. Nature 399: 470–473. 10365959 10.1038/20939

[46] Machens CK (2005) Flexible Control of Mutual Inhibition: A Neural Model of Two-Interval Discrimination. Science 307: 1121–1124. 15718474 10.1126/science.1104171

[47] Miller P, Wang X-J (2006) Inhibitory control by an integral feedback signal in prefrontal cortex: A model of discrimination between sequential stimuli. Proc Natl Acad Sci USA 103: 201–206. 16371469 10.1073/pnas.0508072103 PMC1324991

[48] Deco G, ROlls ET, Romo R (2010) Synaptic dynamics and decision making. Proc Natl Acad Sci USA 107: 7545–7549. 10.1073/pnas.1002333107 20360555 PMC2867686

[49] Barak O, Sussillo D, Romo R, Tsodyks M, Abbott LF (2013) From fixed points to chaos: three models of delayed discrimination. Progress in Neurobiology 103: 214–222. 10.1016/j.pneurobio.2013.02.002 23438479 PMC3622800

[50] Romo R, Hernández A, Zainos A, Salinas E (2003) Correlated neuronal discharges that increase coding efficiency during perceptual discrimination. Neuron 38: 649–657. 12765615 10.1016/s0896-6273(03)00287-3

[51] Romo R, Salinas E (2003) Flutter Discrimination: neural codes, perception, memory and decision making. Nat Rev Neurosci 4: 203–218. 12612633 10.1038/nrn1058

[52] Romo R, Hernández A, Zainos A (2004) Neuronal correlates of a perceptual decision in ventral premotor cortex. Neuron 41: 165–173. 14715143 10.1016/s0896-6273(03)00817-1

[53] Boyan J, Moore AW (1995) Generalization in reinforcement learning: Safely approximating the value function. NIPS: 369–376.

[54] Baird L (1995) Residual algorithms: Reinforcement learning with function approximation. ICML-95: 30–37.

[55] Deubel H, Schneider WX (1996) Saccade target selection and object recognition: Evidence for a common attentional mechanism. Vision Res 36: 1827–1837. 8759451 10.1016/0042-6989(95)00294-4

[56] Schoups A, Vogels R, Qian N, Orban G (2001) Practising orientation identification improves orientation coding in V1 neurons. Nature 412: 549–553. 11484056 10.1038/35087601

[57] Ahissar M, Hochstein S (1993) Attentional control of early perceptual learning. Proc Natl Acad Sci USA 90: 5718–5722. 8516322 10.1073/pnas.90.12.5718 PMC46793

[58] Jiang Y, Chun MM (2001) Selective attention modulates implicit learning. Q J Exp Psychol 54: 1105–1124. 11765735 10.1080/713756001

[59] Moore T, Armstrong KM (2003) Selective gating of visual signals by microstimulation of frontal cortex. Nature 421: 370–373. 12540901 10.1038/nature01341

[60] Roelfsema PR, van Ooyen A, Watanabe T (2010) Perceptual learning rules based on reinforcers and attention. Trends Cogn Sci 14: 64–71. 10.1016/j.tics.2009.11.005 20060771 PMC2835467

[61] Kilgard MP, Merzenich MM (1998) Cortical Map Reorganization Enabled by Nucleus Basalis Activity. Science 279: 1714–1718. 9497289 10.1126/science.279.5357.1714

[62] Richardson RT, DeLong MR (1986) Nucleus basalis of Meynert neuronal activity during a delayed response task in monkey. Brain Res 399: 364–368. 3828770 10.1016/0006-8993(86)91529-5

[63] Peck CJ, Salzman CD (2014) The Amygdala and Basal Forebrain as a Pathway for Motivationally Guided Attention. J Neurosci 34: 13757–13767. 10.1523/JNEUROSCI.2106-14.2014 25297102 PMC4188973

[64] Easton A, Ridley RM, Baker HF, Gaffan D (2002) Unilateral lesions of the cholinergic basal forebrain and fornix in one hemisphere and inferior temporal cortex in the opposite hemisphere produce severe learning impairments in rhesus monkeys. Cereb Cortex 12: 729–736. 12050084 10.1093/cercor/12.7.729

[65] Liu Z, Zhou J, Li Y, Hu F, Lu Y, et al (2014) Dorsal Raphe Neurons Signal Reward through 5-HT and Glutamate. Neuron 81: 1360–1374. 10.1016/j.neuron.2014.02.010 24656254 PMC4411946

[66] Fusi S, Drew PJ, Abbott LF (2005) Cascade Models of Synaptically Stored Memories. Neuron 45: 599–611. 15721245 10.1016/j.neuron.2005.02.001

[67] Friedrich J, Urbanczik R, Senn W (2011) Spatio-Temporal Credit Assignment in Neuronal Population Learning. PLoS Comput Biol 7: e1002092 10.1371/journal.pcbi.1002092 21738460 PMC3127803

[68] Seung HS (2003) Learning in spiking neural networks by reinforcement of stochastic synaptic transmission. Neuron 40: 1063–1073. 14687542 10.1016/s0896-6273(03)00761-x

[69] Izhikevich EM (2006) Solving the Distal Reward Problem through Linkage of STDP and Dopamine Signaling. Cereb Cortex 17: 2443–2452. 10.1093/cercor/bhl152 17220510

[70] Urbanczik R, Senn W (2009) Reinforcement learning in populations of spiking neurons. Nat Neurosci 12: 250–252. 10.1038/nn.2264 19219040

[71] Potjans W, Diesmann M, Morrison A (2011) An Imperfect Dopaminergic Error Signal Can Drive Temporal-Difference Learning. PLoS Comput Biol 7: e1001133 10.1371/journal.pcbi.1001133 21589888 PMC3093351

[72] O’Reilly RC, Frank MJ (2006) Making working memory work: a computational model of learning in the prefrontal cortex and basal ganglia. Neural Comp 18: 283–328. 10.1162/089976606775093909 16378516

[73] Suri RE, Schultz W (1998) Learning of sequential movements by neural network model with dopamine-like reinforcement signal. Exp Brain Res 121: 350–354. 9746140 10.1007/s002210050467

[74] Hoerzer GM, Legenstein R, Maass W (2014) Emergence of complex computational structures from chaotic neural networks through reward-modulated Hebbian learning. Cereb Cortex 24: 677–690. 10.1093/cercor/bhs348 23146969

[75] Williams RJ (1992) Simple statistical gradient-following algorithms for connectionist reinforcement learning. Mach Learn 8: 229–256.

[76] Fremaux N, Sprekeler H, Gerstner W (2013) Reinforcement Learning Using a Continuous Time Actor-Critic Framework with Spiking Neurons. PLoS Comput Biol 9: e1003024 10.1371/journal.pcbi.1003024 23592970 PMC3623741

[77] Zipser D (1991) Recurrent network model of the neural mechanism of short-term active memory. Neural Comp 3: 179–193. 10.1162/neco.1991.3.2.179 31167307

[78] Hochreiter S, Schmidhuber J (1997) Long short-term memory. Neural Comp 9: 1735–1780. 10.1162/neco.1997.9.8.1735 9377276

[79] O’Reilly RC, Hazy TE, Herd SA (2012) The leabra cognitive architecture: how to play 20 principles with nature and win! The Oxford Handbook of Cognitive Science.

[80] O’Reilly RC, Munakata Y (2000) Computational Explorations in Cognitive Neuroscience: Understanding the Mind by Simulating the Brain the MIT Press.

[81] Matsumoto K (2003) Neuronal Correlates of Goal-Based Motor Selection in the Prefrontal Cortex. Science 301: 229–232. 12855813 10.1126/science.1084204

[82] Wallis JD (2007) Orbitofrontal Cortex and Its Contribution to Decision-Making. Annu Rev Neurosci 30: 31–56. 17417936 10.1146/annurev.neuro.30.051606.094334

[83] Luk CH, Wallis JD (2009) Dynamic Encoding of Responses and Outcomes by Neurons in Medial Prefrontal Cortex. J Neurosci 29: 7526–7539. 10.1523/JNEUROSCI.0386-09.2009 19515921 PMC2718717

[84] Parisien C, Anderson CH, Eliasmith C (2008) Solving the problem of negative synaptic weights in cortical models. Neural Comp 20: 1473–1494. 10.1162/neco.2008.07-06-295 18254696

[85] Self MW, Kooijmans RN, Supèr H, Lamme VAF, Roelfsema PR (2012) Different glutamate receptors convey feedforward and recurrent processing in macaque V1. Proc Natl Acad Sci USA 109: 11031–11036. 10.1073/pnas.1119527109 22615394 PMC3390882

[86] Sherman SM, Guillery RW (1998) On the actions that one nerve cell can have on another: distinguishing “drivers” from ‘modulators’. Proc Natl Acad Sci USA 95: 7121–7126. 9618549 10.1073/pnas.95.12.7121 PMC22761

[87] De Pasquale R, Sherman SM (2011) Synaptic Properties of Corticocortical Connections between the Primary and Secondary Visual Cortical Areas in the Mouse. J Neurosci 31: 16494–16506. 10.1523/JNEUROSCI.3664-11.2011 22090476 PMC3233982

[88] Sajikumar S, Korte M (2011) Metaplasticity governs compartmentalization of synaptic tagging and capture through brain-derived neurotrophic factor (BDNF) and protein kinase Mζ (PKMζ). Proc Natl Acad Sci USA 108: 2551–2556. 10.1073/pnas.1016849108 21248226 PMC3038737

